# Multiscale tracking of emulsion dynamics by aggregation-induced emission

**DOI:** 10.1093/nsr/nwaf378

**Published:** 2025-09-09

**Authors:** Jin Wang, Xinyue Liu, Zihe Liu, Yucheng Ma, Shunjie Liu, Jinqing Qu, Ryan T K Kwok, Jacky W Y Lam, Xianhong Wang, Ben Zhong Tang

**Affiliations:** Department of Chemistry, Hong Kong Branch of Chinese National Engineering Research Center for Tissue Restoration and Reconstruction, Department of Chemical and Biological Engineering, Division of Life Science, and State Key Laboratory of Molecular Neuroscience, The Hong Kong University of Science and Technology, Hong Kong 999077, China; Department of Chemistry, Hong Kong Branch of Chinese National Engineering Research Center for Tissue Restoration and Reconstruction, Department of Chemical and Biological Engineering, Division of Life Science, and State Key Laboratory of Molecular Neuroscience, The Hong Kong University of Science and Technology, Hong Kong 999077, China; Key Laboratory of Polymer Eco-materials, Changchun Institute of Applied Chemistry, Chinese Academy of Sciences, Changchun 130022, China; Department of Chemistry, Hong Kong Branch of Chinese National Engineering Research Center for Tissue Restoration and Reconstruction, Department of Chemical and Biological Engineering, Division of Life Science, and State Key Laboratory of Molecular Neuroscience, The Hong Kong University of Science and Technology, Hong Kong 999077, China; Key Laboratory of Polymer Eco-materials, Changchun Institute of Applied Chemistry, Chinese Academy of Sciences, Changchun 130022, China; School of Chemistry and Chemical Engineering, South China University of Technology, Guangzhou 510641, China; Department of Chemistry, Hong Kong Branch of Chinese National Engineering Research Center for Tissue Restoration and Reconstruction, Department of Chemical and Biological Engineering, Division of Life Science, and State Key Laboratory of Molecular Neuroscience, The Hong Kong University of Science and Technology, Hong Kong 999077, China; Department of Chemistry, Hong Kong Branch of Chinese National Engineering Research Center for Tissue Restoration and Reconstruction, Department of Chemical and Biological Engineering, Division of Life Science, and State Key Laboratory of Molecular Neuroscience, The Hong Kong University of Science and Technology, Hong Kong 999077, China; Key Laboratory of Polymer Eco-materials, Changchun Institute of Applied Chemistry, Chinese Academy of Sciences, Changchun 130022, China; Department of Chemistry, Hong Kong Branch of Chinese National Engineering Research Center for Tissue Restoration and Reconstruction, Department of Chemical and Biological Engineering, Division of Life Science, and State Key Laboratory of Molecular Neuroscience, The Hong Kong University of Science and Technology, Hong Kong 999077, China; Guangdong Basic Research Center of Excellence for Aggregate Science, School of Science and Engineering, The Chinese University of Hong Kong (Shenzhen), Shenzhen 518172, China

**Keywords:** *in situ* monitoring, film formation, ionic polymers, interface dynamics, carbon dioxide fixation

## Abstract

The inherent multiscale dynamics of materials necessitate synchronous cross-scale investigation, while traditional multiplatform methods always encounter low operational efficiency and analytical uncertainty. Herein, we try to address these limitations by developing a unified monitoring strategy through aggregation-induced emission fluorogens (AIEgens), whose high-contrast photoluminescence (PL) activation triggered by restricted intramolecular motion (RIM) enables multiscale correlation. Molecular-level analysis with AIEgens reveals ultrasensitive PL transitions during emulsion-to-film evolution, quantifying dynamic shifts from movement-free to being restricted by the polymer chain. This molecular responsiveness enables concurrent tracking of particle coalescence and phase transitions at the microscopic level. At the same time, the AIEgen-assisted monitoring platform reveals drying dynamics at the macroscopic scale with high contrast and precision. Through dual feasibility verification in the laboratory and industry, the methodology integrates molecular conformational dynamics, microscale structural rearrangements and macroscale morphological evolution into a single optical framework, which effectively circumvents the platform-dependent analytical limitations. With polymer emulsion as a case study, we provide a generalizable platform for investigating multiscale dynamics in complex material systems, bridging critical gaps between scale-specific observations and holistic process interpretation.

## Teaser

An AIE platform, proved with polymer emulsions, unifies multiscale dynamics to connect scale-specific observations with holistic process interpretation.

## INTRODUCTION

The complicated multiscale dynamics inherently exist within matter. To elucidate the interconnections between these scales, researchers always investigate from various scales to obtain comprehensive insights [1–3]. This approach is essential for uncovering fundamental mechanisms and governing principles. For example, in the study of cellular signal transduction, researchers always analyze the binding interactions between signal molecules and receptors at the molecular scale, trace intracellular signaling pathways at the microscopic scale, and further explore how these signals influence physiological functions at the macroscopic level [4,5]. Such investigations necessitate the integration of molecular, microscopic and macroscopic insights to achieve a holistic understanding of cellular response mechanisms. This multiscale perspective is widely feasible across life sciences, including ordinary phenomena such as gene expression, immune responses and organ development [6–8]. Similarly, in materials science, multiscale research has emerged as a pivotal paradigm for revealing the structure–property relationships and dynamic evolution mechanisms of materials. Research across different scales always offers unique insights. Molecular behaviors dictate microscopic structures, which in turn influence macroscopic properties. Conversely, changes in macroscopic properties can often be traced back to transformations at the microscopic or molecular levels [9–11]. Bridging these scales into a unified framework is critical for systematically understanding the dynamic internal mechanisms of materials.

**Figure 1.**
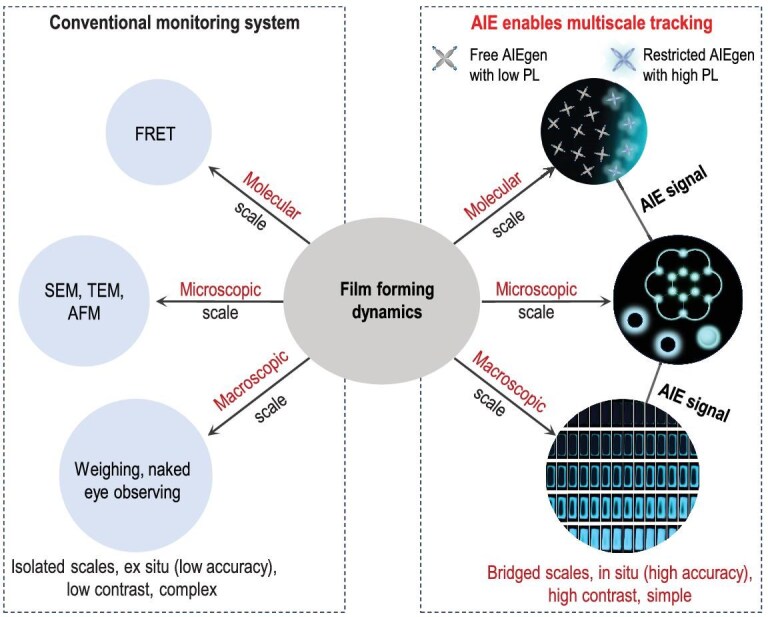
Multiscale tracking strategy integrating molecular, microscopic and macroscopic approaches, comparing conventional (left) and AIE-enabled (right) monitoring systems.

Benefiting from water as a green solvent for dispersion, polymer emulsions represent one of the most widely utilized polymer materials in coatings, adhesives, binders and the printing inks industry [12–14]. In 2023, global consumption of polymer emulsion surpassed 3 million tons, with a market reaching up to $50 billion. Considering that the purpose of an emulsion is to provide a high-performance coating film, its film formation is equally important as its inherent properties. However, investigating polymer emulsion film formation remains challenging due to its complex phase-separated structure and the lack of real-time dynamic monitoring platforms for the wet system. Film formation involves intricate multiscale dynamic processes such as water evaporation, particle–particle contact and fusion, and polymer molecular diffusion [15–17]. These processes span molecular, microscopic and macroscopic scales, necessitating a comprehensive understanding from a multiscale perspective. To study them, researchers have employed diverse methodologies tailored to specific scales (Fig. 1). At the molecular scale, fluorescence resonance energy transfer (FRET) has been used to monitor the fusion of polymer molecules through energy transfer between donor and acceptor molecules [18,19]. At the microscopic scale, electron-based techniques such as scanning electron microscopy (SEM) and transmission electron microscopy (TEM), as well as scanning probe methods like atomic force microscopy (AFM), have been developed to study particle–particle interactions during film formation [20–22]. At the macroscopic scale, the film-formation progress is often monitored by weighing, or judged using sensory means of visual inspection or tactile evaluation. While these methods are feasible in some sense, their limitations, such as specific sample requirements, low contrast and inability to provide real-time, high-precision dynamic tracking, constrain their feasibility. Moreover, the reliance on diverse technologies introduces various challenges, including high investigation, complex sample preparation, lengthy research cycles and uncertainties in data processing and integration across scales. A single technology capable of bridging molecular, microscopic and macroscopic scales would significantly enhance the convenience and reliability of studying polymer emulsion film formation.

Fluorescence-based technologies, encompassing molecular sensing, microscopic imaging and macroscopic detection, hold the potential to meet this challenge. These methods integrate three key components of fluorescent probe, fluorescence microscopy (FM) and fluorescence spectrometry [23–25]. While fluorescence spectrometry is a mature platform and FM has achieved high resolutions up to 20–50 nm, developing a suitable fluorescent probe is critical for realizing this multiscale approach [26–29]. Aggregation-induced emission (AIE) has recently emerged as a cutting-edge topic in materials science [30–33]. Unlike the traditional phenomenon of aggregation-caused quenching, AIE fluorogens (AIEgens) are photoluminescent (PL) inactive in the solution state due to high molecular motion freedom and motion-induced fast non-radiative decay. Upon aggregation, however, their PL is activated due to the restriction of intramolecular motion (RIM) [34–39]. As a novel fluorescent probe, AIEgens exhibit unparalleled advantages for real-time, *in situ* monitoring of dynamic processes, providing high contrast and precision [40–42]. These merits make AIEgens promising candidates for bridging multiscale dynamics in complex systems. In this study, we employ a multiscale monitoring approach leveraging AIE technology to gain deeper insights into the film-forming dynamics of complex polymer emulsions. By validating the feasibility of integrating molecular, microscopic and macroscopic observations through a single fluorescence-based platform of AIE, this work aims to establish a robust framework for studying multiscale dynamics (Fig. 1).

## RESULTS AND DISCUSSION

### Differential fluorescence imaging

**Figure 2.**
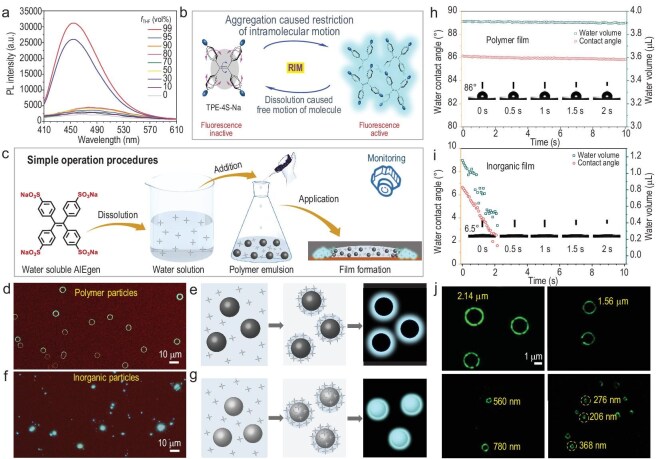
AIE properties of TPE-4S-Na and FM visualization of polymer and inorganic particles. (a) PL spectra of TPE-4S-Na in THF/water mixtures with different THF fractions (*f*_THF_). Concentration = 10^−5^ M, *λ*_ex_ = 405 nm. (b) Schematic of TPE-4S-Na transitioning from a freely moving dissolved state to a movement-restricted aggregate state. (c) Workflow for sample preparation. (d) FM image of CO_2_-PUD particles stained with TPE-4S-Na (average size: 4.0 μm). (e) Schematic drying mechanism for polymer emulsions. (f) FM image of CC powder stained with TPE-4S-Na. (g) Proposed drying dynamics for inorganic particles in aqueous media. (h) The water contact angle and water volume on polymer film versus contact time. (i) The water contact angle and water volume on the inorganic film versus contact time. (j) Super-resolution FM images (SIM mode on Elyra-7) of CO_2_-PUD particles with varying sizes. All samples (0.05 wt% particle concentration) contained 5 wt% TPE-4S-Na fluorescent probe relative to solid content. Scale bars: 10 μm in (d) and (f); 1 μm in (j).

In recent years, CO_2_-based polyurethane dispersion (CO_2_-PUD) has emerged as a high-performance polymer emulsion, exhibiting superior mechanical strength, hydrolysis resistance and tunable properties [43,44]. Furthermore, utilizing water as a solvent and CO_2_, a greenhouse gas, as a raw material positions CO_2_-PUD as an eco-friendly material that contributes to carbon fixation while offering novel opportunities for the materials industry (Schemes S1–3 in the Supplementary data). Sodium 4,4′,4′′,4′′′-(ethene-1,1,2,2-tetrayl)tetrabenzenesulfonate (TPE-4S-Na), a derivative of the widely studied AIEgen of tetraphenylethylene (TPE), features four sodium sulfonate groups, which impart excellent water solubility to it (Figs S1 and S2). As demonstrated in Fig. 2a, when 99 vol% of the good solvent of water was replaced with the poor solvent of tetrahydrofuran (THF), a 14-fold enhancement in PL intensity was observed, confirming the pronounced AIE behavior of TPE-4S-Na. In aqueous solution, the free molecular motion of the AIEgen dissipated excitation energy through non-radiative decay, leading to weak PL (Fig. 2b). Upon addition of THF, TPE-4S-Na transitioned from a dissolved solution state to an aggregate state, restricting molecular motion and suppressing non-radiative decay, thereby significantly enhancing PL. The good water solubility of TPE-4S-Na ensured a robust AIE response. Additionally, its fluorescence can be efficiently excited at 365 nm, with an emission spectrum spanning 380–650 nm, covering the visible range (Figs S3–S10). These properties established TPE-4S-Na as a promising candidate for industrial fluorescence applications.

Figure 2c illustrates the experimental procedure, wherein TPE-4S-Na was first dissolved in water and subsequently incorporated as a fluorescent probe into CO_2_-PUD. The emulsion complex was applied onto a glass slide and then analyzed using FM. As shown in Fig. 2d, bright PL was observed at the edges of CO_2_-PUD particles, while the central regions remained dark, indicative of a negative staining effect of TPE-4S-Na on polymer particles (Figs S11 and S12). In contrast, the body of calcium carbonate (CC), an inorganic particle, exhibited bright fluorescence under FM, demonstrating a positive staining effect (Fig. 2f, Figs S13–S15). This differential staining behavior was attributed to the difference in hydrophilicity between these two kinds of particles. The carboxyl groups in CO_2_-PUD facilitated its dispersion in water; however, the limited hydrophilic components were insufficient to overcome its inherently hydrophobic polyurethane backbone, as evidenced by a relatively higher water contact angle of 86° (immediately measured, Fig. 2h). The contact angle and water volume of the drop on the dry film of CO_2_-PUD remained unchanged during the 10 s immersion on the film surface. Conversely, CC, as an ionic salt, exhibited high polarity and hydrophilicity, with a significantly lower water contact angle of 6.5° (Fig. 2i, Figs S16 and S17). The contact angle and volume of the water drop showed a sharp reduction during the 0.5 s immersion on the film surface, further verifying the hydrophilic nature of CC.

As depicted in Fig. 2e, during the drying process, TPE-4S-Na in water migrated toward the polymer particle surfaces as water evaporated. The hydrophobic nature of CO_2_-PUD inhibited water from wetting the particle surfaces, confining the water and TPE-4S-Na to the vicinity of the particles. Upon complete water evaporation, the molecular motion restriction of TPE-4S-Na resulted in strong PL at the particle contours, while the interiors remained non-luminescent. In contrast, the hydrophilicity of CC allowed water to wet the particle surfaces smoothly during water evaporation (Fig. 2g), enabling TPE-4S-Na to interact with the particle body, thereby producing a bright PL signal indicative of positive staining. To achieve higher imaging resolution, CO_2_-PUD particles of varying sizes were analyzed using structured illumination microscopy (SIM), a super-resolution fluorescence imaging mode (Fig. 2j, Fig. S18). This technique enabled the imaging of particle sizes ranging from 206 nm to 2.14 μm, encompassing the size range of most industry polymer emulsions. Additionally, super-resolution imaging of CO_2_-PUD blended with CC, talcum powder (TP) and titanium dioxide (TD) demonstrated that negatively stained CO_2_-PUD particles could be easily distinguished from such complex systems (Figs S19–S26), further showcasing the practical utility of TPE-4S-Na in industrial applications.

### Molecular scale monitoring

**Figure 3.**
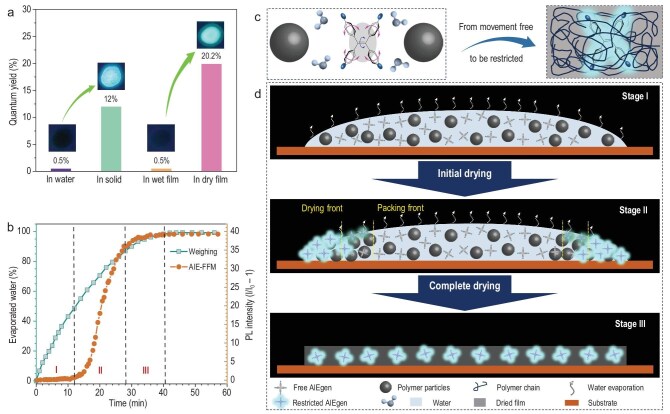
Molecular-scale monitoring of film formation. (a) QY measurements and UV-irradiated photographs of TPE-4S-Na in distinct states. (b) Temporal profiles of water evaporation and normalized PL intensity (*I/I*_0_ − 1) during drying (CO_2_-PUD: 38 wt% solid content; 5 wt% TPE-NS-Na probe in solid content; dried at 25°C ± 3°C). (c) Proposed transition mechanism of TPE-4S-Na from a freely moving dissolved state to a movement-restricted state within the polymer matrix. (d) Schematic illustrating AIE-FFM for monitoring emulsion-film formation.

The film-forming dynamics of polymer emulsions play a critical role in determining the properties of the resulting dried film, making this process a subject of significant research interest [17,45–49]. However, the complexity of emulsion structures and the lack of an effective platform for tracking dynamic processes in wet systems have posed considerable challenges. To date, few techniques have been developed that are suitable for real-time monitoring of film formation both in academic and industrial contexts. In the solution state, TPE-NS-Na exhibited weak PL with a low quantum yield (QY) of 0.5%, while transitioning to the solid state after drying resulted in bright PL with a significantly higher QY of 12% (Fig. 3a). A similar phenomenon was observed when TPE-4S-Na was incorporated into CO_2_-PUD. In the dissolved state in emulsion, weak PL with a QY of 0.5% was recorded, whereas drying the emulsion produced a bright PL with a QY of 20.2%. Inspired by the restriction of the RIM mechanism and the transition of TPE-4S-Na from a free-movement state to a restricted state during drying, TPE-4S-Na was employed as a fluorescent probe to monitor the film-forming dynamics of polymer emulsions through an AIE-based film-formation monitoring (AIE-FFM) strategy. As shown in Fig. 3b, the drying process began with rapid water evaporation in the early stage, which finally slowed and was complete after 41 min. In addition to the conventional weighing method, real-time PL monitoring was performed using a fluorescence spectrometer, characterizing the drying process into three distinct stages. During the initial stage (0–11 min, stage I), water evaporation occurred in other areas of the wet film, with the monitored region remaining wet, resulting in no significant change in PL intensity. In stage II (11–27 min), rapid water evaporation caused TPE-4S-Na to transition from a free-movement state to a restricted state, leading to a sharp increase in PL intensity. The final stage (27–41 min, stage III) was characterized by a slower increase in PL intensity, eventually reaching a constant peak value, indicating the complete drying of the film. The transition of AIEgen molecules from a dissolved, freely moving state to a dried, movement-restricted state represents a molecular-level change. This behavior can be simply monitored by fluorescence intensity, thereby achieving molecular scale monitoring.

The mechanism of AIE-FFM is illustrated in Fig. 3c and d. Initially, TPE-4S-Na was dissolved in the wet film before drying. As drying progressed, water evaporation began at the edges of the film, forming a dry region where TPE-4S-Na transitioned from a dissolved state to being confined by polymer chains, resulting in strong PL. Conversely, the central area of the film remained wet, allowing free molecular motion of the AIEgens, which quenched PL. An intermediate region with moderate PL intensity was observed between the wet and dry areas, indicating the accumulation and packing of polymer particles as most of the water evaporated. The final stage, marked by the complete evaporation of water, led to uniform bright PL across the entire film, signaling the end of the drying process. Compared to the traditional weighing method, AIE-FFM offers several advantages. First, its *in situ* molecular-level sensitivity enables the precise identification of all three drying stages, providing more detailed insights into the film-forming process. Second, unlike the macroscopic monitoring offered by weighing, AIE-FFM facilitates real-time monitoring of specific localized regions, offering higher spatial resolution. Additionally, as only the dried regions emit fluorescence, this technique provides a more accurate insight into the true film-forming dynamics. These advantages make AIE-FFM a promising strategy for the real-time monitoring of polymer emulsion film formation in both research and industrial applications.

### Microscopic-scale monitoring

**Figure 4.**
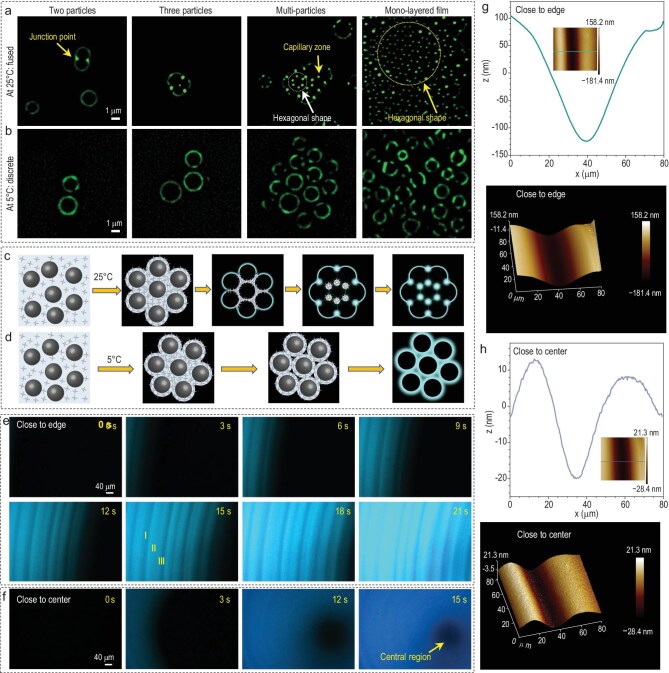
Microscopic-scale monitoring of film formation. (a and b) Fusion dynamics of CO_2_-PUD particles (0.05 wt% emulsion, 5 wt% TPE-NS-Na probes in solid content) under FM with varying particle numbers at 25°C and 5°C, respectively. (c and d) Proposed mechanistic model of particle coalescence at 25°C and 5°C, respectively. (e and f) FM images near the edge (e) and center (f) of wet films at different drying times (5 wt% CO_2_-PUD, 5 wt% TPE-NS-Na probes in solid content; 1.24 mm × 0.88 mm field). (g and h) The depth-value curves and AFM images near the edge (g) and the center (h) of the dry film. Scale bars: 1 μm in (a) and (b); 40 μm in (e) and (f). All experiments were conducted at 25°C ± 3°C.

In many cases, film formation defects cannot be observed at the macroscopic level and require further investigation on the microscale to assess the quality of the film. These microscopic defects can directly impair the mechanical properties and protective performance of the coating. Therefore, a multiscale approach is essential for comprehensively evaluating the film-formation process. Benefiting from precise *in situ* sensing towards the transition of TPE-4S-Na from a free-movement state in solution to a state restricted by the polymer matrix, AIE-FFM provided a precise monitoring approach for film-forming dynamics at the molecular level. Monitoring the dynamic process at the microscopic level facilitates the investigation of particle interactions and microphase transitions, thus playing a vital role in studying the film-forming mechanisms. The mechanical property of the polymer is always influenced by environmental temperature, making the film-forming temperature a critical factor in determining the drying efficiency of the polymer emulsion [17,45]. Here, CO_2_-PUD containing TPE-4S-Na was coated onto a glass slide, and particles with varying numbers were dried at 25°C or 5°C. Observations made under super-resolution FM revealed distinctly different particle fusion behaviors. Upon drying at 25°C, a notable fusion behavior was observed between adjacent particles (Fig. 4a, Figs S28 and S29). Specifically, weak PL was detected around the periphery of the two particles, while no fluorescence was emitted from the fused regions between them. Interestingly, strong PL was observed at the junction points between two particles, attributed to the significant aggregation of TPE-4S-Na during drying. A similar phenomenon was observed when the number of particles increased to three. As the number of particles further increased to a multiparticle system, we observed hexagonally distributed capillary regions at the points of particle fusion [46], resulting from the selective aggregation of TPE-4S-Na in these areas during drying. When the system was further scaled up to encompass the entire monolayer film, the intact hexagonal-shaped capillary regions were captured. In contrast, Fig. 4b illustrates that the particles dried at 5°C did not exhibit similar fusion behaviors. Instead, they displayed distinct, separated and intact circular PL surrounding their peripheries.

The difference in PL intensity across different regions signified the residual traces left behind by the drying process of emulsion particles, which contributed to a deeper understanding of the particle interaction dynamics during film-forming at 25°C. As illustrated in Fig. 4c, the evaporation of water induced the aggregation of water, TPE-NS-Na and emulsion particles by the surface tension of water. Subsequent evaporation resulted in the initial drying of the outer regions of the particles, accompanied by the lighting up of PL and the progressive proximity of emulsion particles. At this stage, water accumulated in the gaps between particles and evaporated continuously in the capillary zone. The capillary forces generated from the evaporation of water in the capillary zone continuously drove the deformation of surrounding emulsion particles, thus promoting further particle fusion. During this process, water accumulated from the adjacent area of particles towards the capillary zone. Due to the intense and prolonged water evaporation in the capillary zone, TPE-4S-Na was driven to continuously accumulate towards this area, resulting in a significantly brighter fluorescence than other regions after complete drying. As water evaporated at 5°C, the aggregation was also proposed, as in Fig. 4d. However, the low temperature prevented the deformation of emulsion particles when they came into proximity. Further evaporation of water resulted in the immobilization of TPE-4S-Na surrounding each emulsion particle, leading to the preserved spherical shape of particles, distinguishing them from those dried at 25°C. Benefiting from the high sensitivity of TPE-4S-Na towards micro-environment changes and its negative staining effect on polymer particles, the particle interaction dynamics during film formation could be monitored from a microscopic perspective.

After examining the drying behavior of particle-scale and monolayer CO_2_-PUD particles, the study was extended to a multilayer thin-film emulsion system. Figure 4e depicts the film-formation process in a region near the edge of a wet film, with a monitored area of 1.24 mm × 0.88 mm. At 0 s, TPE-4S-Na remained dissolved, and no PL was detected. By 3 s, fluorescence began to emerge on the left side, indicating the complete evaporation of water in this region. As drying progressed, the fluorescent region expanded from left to right, eventually encompassing the entire monitoring area after 21 s. Notably, the drying process exhibited an arc-shaped progression from the outer to the inner area, leaving alternating bright and dark rings at 15 s, indicative of the ‘coffee ring effect.’ This phenomenon resulted from surface tension-driven evaporation edges [47–49]. The evaporation edge caused water from adjacent regions (e.g. region II) to diffuse toward the edge (region I), carrying TPE-4S-Na and polymer particles. This migration led to the formation of a brighter fluorescent ring (region I) with greater thickness, while region II exhibited fewer particles and AIEgens, drying more quickly and appearing as a darker ring. Interestingly, the central region displayed significantly lower PL intensity than other regions (Fig. 4f), suggesting that water evaporation caused outward diffusion of TPE-4S-Na from the inner areas. Thickness differences between regions I and II in the area close to the edge reached 227 nm (Fig. 4g), while that of the region close to the center showed a much lower value of 33 nm (Fig. 4h). Based on the information provided by AFM, the signal intensity of PL accurately reflected the actual thickness information of the coating. The high contrast in PL intensity of TPE-4S-Na before and after drying enables real-time monitoring of the transition dynamics of CO_2_-PUD from a wet emulsion to a fully dried film, providing a detailed microscopic perspective of the film-formation process.

### Macroscopic-scale monitoring

**Figure 5.**
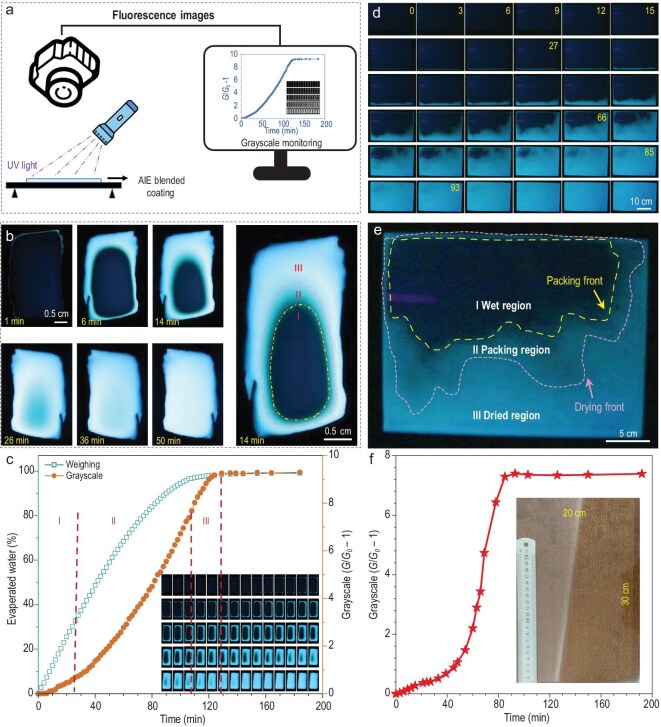
Macroscopic-scale monitoring of film formation. (a) Customized AIE-FFM imaging setup. (b) Time-dependent weak-UV (4 mW/cm²) images of drying CO_2_-PUD films (38 wt% solid content, 1 wt% TPE-NS-Na probe in solid content). (c) Correlation between water loss and normalized grayscale (*G/G*_0_ − 1) over time (inset: weak-UV images). (d) Weak-UV visualization of drying waterborne wood coatings (38 wt% solids, 0.5 wt% TPE-NS-Na probe in solid content) on substrates at specified intervals. (e) Weak-UV visualization of drying waterborne wood coatings at 66 min. (f) Grayscale evolution kinetics for wood coatings (inset: daylight image of dry film). Scale bars: 0.5 cm in (b); 10 cm in (d); 5 cm in (e). All films were dried at 25°C ± 3°C.

Due to the high sensitivity and accuracy of TPE-4S-Na in detecting microenvironmental dynamics at the molecular scale, along with the strong contrast between wet and dried regions under FM, AIE-FFM demonstrated excellent reliability in real-time monitoring of dynamic processes at the microscopic level. This approach provided a novel and effective method for studying the mechanisms underlying the film-forming dynamics of polymer emulsions. With its simplicity and significant potential for practical applications, AIE-FFM was also extended to monitor film formation at the macroscopic level. To address the relatively high costs of acquisition and maintenance, and the environmental constraints of using a fluorescence spectrometer, the monitoring platform was modified. Inspired by the correlation between fluorescence brightness and grayscale values, grayscale analysis was adopted as a substitute for PL intensity in monitoring film-forming dynamics. As shown in Fig. 5a, a modified platform for AIE-FFM was developed, where fluorescence images of the entire wet film under UV light were captured in real time using a camera and then converted into grayscale curves.

Figure 5b displays the fluorescence images of a CO_2_-PUD film at various drying times. At 1 min, strong PL was observed at the edges, indicating complete drying in this region. As drying progressed, the illuminated area expanded inward and eventually covered the entire film after 50 min, signaling the completion of the film-forming process. At 14 min, the drying film was divided into three distinct regions by the packing front and drying front. Region I displayed almost no PL, representing a wet area with no drying. Region II exhibited weak PL, which corresponded to the packing of emulsion particles. Region III showed strong PL, indicating the dried area. Simultaneously, weighing data, fluorescence images and grayscale values were collected for the CO_2_-PUD film, as shown in Fig. 5c. The drying process began with rapid water evaporation, which gradually slowed and was completed at 129 min. The PL intensity evolution was divided into three stages according to the fluorescence images and grayscale curves. PL intensity grew slowly during the first 30 min (Stage I). Between 30 and 110 min (Stage II), a rapid increase in PL intensity was observed, followed by a slower increase from 110 to 129 min (Stage III), when the PL intensity plateaued, marking the complete drying of the film. The monitoring results with different addition amounts of TPE-4S-Na were further presented in Figs S30 and S31.

Compared to the weighing method, AIE-FFM provides a more precise characterization of the drying process by segmenting it into three distinct stages based on grayscale variations, offering results that more accurately reflect the actual film-forming dynamics. Additionally, AIE-FFM fully exploits the differences in PL behavior between TPE-4S-Na in solution and restricted states, enabling high-contrast imaging to differentiate wet and dried areas. Furthermore, AIE-FFM allows real-time monitoring of both the entire film and specific localized regions. Importantly, this method does not require specialized monitoring conditions, expensive equipment, or strict constraints on sample size, substrate or form, making it highly adaptable and user-friendly for practical applications in the coatings industry.

### Practical application in the coatings industry

Building on the molecular- and microscopic-scale investigations, AIE-FFM was successfully extended to macroscopic monitoring, offering an intuitive approach to understanding the film-forming dynamics of polymer emulsions. To demonstrate practical feasibility, AIE-FFM was further applied in the coatings industry. Waterborne wood coatings, which have gained significant traction in the past decade due to their environmental benefits, were selected as a model system. TPE-4S-Na was incorporated into an industrial-grade waterborne wood coating, sprayed onto a 30 cm × 20 cm wooden board, and monitored using the AIE-FFM. As shown in Fig. 5d, fluorescence images captured at different drying times revealed that drying began in the lower region of the board and gradually progressed upward, eventually covering the entire surface. The fluorescence image at 66 min (Fig. 5e) illustrated the division of the coating into wet region, the packing region and the dried region, separated by the packing front and drying front. Figure 5f shows the daylight image of the dried wooden board, alongside the grayscale–time curve for the drying process. Analysis of the fluorescence images and grayscale curve indicated that the coating dried slowly during the first 27 min, followed by a rapid drying phase from 27 to 85 min. In the final stage, PL intensity increased gradually and reached a peak value at 93 min, marking the completion of the film-forming process. The successful application of AIE-FFM to monitor waterborne wood coatings demonstrated its practicality for industrial use. Beyond fundamental research, AIE-FFM was shown to meet the demands of real-world applications in the coatings industry. Moreover, the feasibility of this strategy was further explored in other systems, including anionic, non-ionic and cationic polyurethane emulsions, as well as polyacrylate emulsions, one of the most widely used types of industrial coatings (Figs S32–S34).

## CONCLUSION

Multiscale analysis of material systems offers researchers comprehensive insights and elucidates the interconnections between these scales, yet the inherent complexity of integrating disparate methodologies across dimensional regimens often introduces experimental uncertainties. This study addresses this challenge through a unified AIE-enabled strategy. At the molecular level, the RIM mechanism of TPE-4S-Na enabled ultrasensitive detection of dynamic transitions between movement-free and restricted states, evidenced by pronounced PL contrast during emulsion-to-film evolution. Leveraging this molecular responsiveness, real-time tracking of particle interactions and phase transitions was achieved at the microscopic scale, establishing a robust framework for elucidating film-formation dynamics. The systematic integration of molecular and microscopic observations facilitated seamless macroscopic implementation of AIE-FFM, which provided unprecedented visualization of drying dynamics in polymer emulsions. The developed multiscale platform demonstrates the viability of bridging molecular, microscopic and macroscopic phenomena through a singular AIE-based technology. Notably, the optimized AIE-FFM system exhibited operational simplicity, high precision and broad sample compatibility, requiring minimal infrastructure investment. Practical validation in waterborne wood coatings confirmed its dual utility, as a fundamental research tool for *in situ* tracking of microdynamic processes and as an industrial solution for monitoring film-forming behaviors. This work establishes a paradigm for multiscale interrogation of complex material systems, highlighting the transformative potential of AIE technologies in bridging cross-scale analyses. Analyzing the evolution of dynamic processes across different scales of molecular, microscopic and macroscopic facilitates the establishment of a systematic understanding of these processes. Leveraging the advantages of AIE technology in monitoring dynamic processes, this study connects monitoring across various scales using a unified AIE signal. This approach offers new insights and methodologies for the monitoring of dynamic processes in fields such as life sciences, including tumor metastasis, neural signal transmission and wound healing, as well as in materials science, encompassing battery charging and discharging, and metal corrosion.

## METHODS

### Fluorescence microscopic imaging

#### Differential FM imaging

To prepare the AIEgen solution 1, 0.1 g of TPE-4S-Na was dissolved in 10 mL of deionized water. For the polymer emulsion sample, 0.01 g of CO_2_-PUD (38 wt%) was diluted to a concentration of 0.05 wt%. After that, 0.019 mL of AIEgen solution 1 was added to the diluted CO_2_-PUD (5 wt% in solid), giving the polymer emulsion sample. Inorganic pigment (0.1 g) or filler was added to 5 mL of water under stirring, and the mixture was then diluted to a concentration of 0.05 wt%, and 0.019 mL of AIEgen solution 1 was added, leading to the formation of the inorganic emulsion sample. All of the obtained emulsion samples were spin-coated onto glass slides and finally subjected to FM imaging under an Upright Biological Microscope Ni-U, with 330–385 nm UV light as an exciting light source, or under Zeiss Elyra-7 SIM mode with 405 nm UV light.

#### Differential FM imaging in the coatings industry

A TD emulsion was prepared by dispersing 5 g of TD powder in 20 mL of water under stirring. The CC and TP emulsions were prepared with the same procedure. Next, 3 g of the TD emulsion was added to 10 g of CO_2_-PUD under stirring, and 3 g of the CC and TP emulsions were further added. Next, 0.1 g of blended emulsion was diluted to a concentration of 0.2 wt% using water, and 0.5 mL of AIEgen solution 1 was added for final fluorescence imaging.

### Monitoring of film formation

#### At the molecular level

To prepare the AIEgen solution 2, 0.5 g of TPE-4S-Na was dissolved in 5 mL of deionized water. For the monitoring sample, 0.19 mL of the AIEgen solution was added to 1 g of CO_2_-PUD. The resulting mixture was used for monitoring purposes. To initiate the monitoring process, the sample was dropped onto a quartz plate. The drying process of the wet film was immediately monitored using dynamic PL measurements at 448 nm with excitation by 365 nm UV light. To prevent possible photobleaching caused by continuous exposure to UV light during the monitoring period, the excitation light was intermittently shielded, resulting in discontinuous signals.

#### At the microscopic level

CO_2_-PUD (0.1 g) was diluted to a concentration of 5 wt% using water. Next, 0.038 mL of AIEgen solution 2 was added to the diluted CO_2_-PUD. For the monitoring process, a small drop of the sample was coated onto a glass slide. The wet film was immediately subjected to FM to monitor the film-forming process. In order to observe different regions of the wet film in real-time, fixed-sized regions of 1.24 mm × 0.88 mm were selected at various positions, as illustrated in Fig. 4e and f. Furthermore, the sample was further diluted to a concentration of 0.05 wt% and coated onto a glass slide. The coated slide was then dried at 25°C, and finally monitored under super-resolution FM, as shown in Fig. 4a and b.

#### At the macroscopic level

AIEgen solution 2 (0.38 mL) was added to 1 g of CO_2_-PUD. For the monitoring process, 0.2 mL of the sample was coated onto a glass slide and immediately subjected to the monitoring platform, as depicted in Fig. 5a. The entire drying process was recorded using a camera under UV light with a wavelength of 365 nm. The captured images were then processed using ImageJ software to read the grayscale information.

#### Application in industry

To realize the practical application in the coatings industry, an industrialized wood coating was selected as the study object. Similarly, 2.5 g of TPE-NS-Na was dissolved in 15 g of deionized water, and then added to 100 g of the industrialized waterborne wood coating (provided by Carpoly Chemical Group Co., Ltd.). Subsequently, the sample was spray-coated onto a primed wood board (30 cm × 20 cm). The coated board was immediately subjected to the monitoring platform, images as well as the grayscale changes, drying film formation, were monitored using the previously described methods.

## Supplementary Material

nwaf378_Supplemental_File
