# Supplementary material for: Multiscale tracking of emulsion dynamics by aggregation-induced emission
Source: Natl Sci Rev. 2025 Sep 9;12(11):nwaf378. doi: 10.1093/nsr/nwaf378 (PMC12581895; doi:10.1093/nsr/nwaf378)
Supplement: nwaf378_Supplemental_File [file nwaf378_supplemental_file.pdf]

Supporting Information

Multiscale Tracking of Emulsion Dynamics by Aggregation-Induced Emission

Jin Wang, Xinyue Liu, Zihe Liu, Yucheng Ma, Shunjie Liu, Jinqing Qu, Ryan T. K. Kwok, Jacky W. Y. Lam*, Xianhong Wang* and Ben Zhong Tang**

1. Materials

Tetraphenylethylene (TPE, 97%), concentrated Sulfuric Acid (95%), Isophorone diisocyanate (IPDI), Triethylamine (TEA), di-n-butyl tin dilaurate (DBTDL), 2,2-Bis(hydroxymethyl)propionic acid (DMPA), Ethylene glycol (EDO). All the above-mentioned chemicals were purchased from Energy Chemistry, Sigma-Aldrich, and TCI and used directly without further purification. The 1,1,2,2-tetra (4-carboxyphenyl)ethylene (TPE-4COOH, 97%) was purchased from Energy Chemical and neutralized with Sodium hydroxide to obtain TPE-4COONa. All solvents were purchased from VWR Chemicals Corp. The Changchun Institute of Applied Chemistry supplied the CO_2-polyol. The pigments of Titanium dioxide, Calcium carbonate, and Talcum powder, as well as industrialized polymer emulsion and waterborne wood coatings, were provided by Carpoly Chemical Group Co., Ltd.

2. Synthesis

TPE-4S-Na: The TPE-4S-Na was synthesized according to the reported route.[1, 2] A 0.5g sample of TPE was added to 5 mL of Sulfuric Acid and stirred for 3 h at 110 °C. The reaction mixture was poured into 100 mL of EA and filtered to get white powder of TPE-4S with a yield of 98%. The TPE-4S was dissolved in 3 g of water, and a quadruple equivalent of sodium hydroxide was added. The solution was then dropped into 100 mL of THF and filtered to obtain a white powder of the final product, TPE-4S-Na.

CO_2-WPU (anionic): The CO_2-WPU was synthesized according to a modified acetone process,[3] where THF was used as solvent. Under a nitrogen atmosphere, 60 g of CO_2-polyol was added to a 1000-mL three-necked round-bottom flask, which was kept in an oil bath and equipped with a mechanical stirrer, a nitrogen inlet, and a condenser. Once the reaction temperature reached 65°C, 15 g of IPDI and 20 mg of DBTDL were added to the reaction mixture. 3 g of DMPA was added after all OH in the reaction medium was consumed, and 10 g of IPDI was added when NCO in the reaction medium was undetectable. Finally, 4 g of EDO was introduced into the reaction system to prepare high molecular weight polyurethane, where the total NCO/OH ratio was maintained at 1/1.05 (in mole). Then, 1.8 g TEA was added to neutralize the mixture, and 250 g deionized water was added dropwise under vigorous stirring to get the neutralized PU dispersion. After stirring for about 3 h, the THF was removed by rotary evaporator at 50 °C water bath under vacuum to collect the CO_2-WPU. The DMPA content was adjusted for different particle-sized emulsions.

Nonionic CO_2-WPU: Under a nitrogen atmosphere, 4 g of CO_2-polyol and 2 g of PEG were introduced into a 250-mL three-necked round-bottom flask equipped with a mechanical stirrer, a nitrogen inlet, and a condenser. The flask was placed in an oil bath and heated to 70 °C. At this temperature, 3 g of IPDI and 5 mg of DBTDL were added to the reaction mixture. Subsequently, 0.15 g of TMP and 0.47 g of EDO were added, followed by an additional 0.1 g of EDO to facilitate the formation of high molecular weight polyurethane. After the reaction, 50 g of deionized water was gradually added dropwise under vigorous stirring to form the CO_2-WPU dispersion. The mixture was stirred continuously for approximately 3 hours. Finally, the THF solvent was removed by rotary evaporation at 50 °C in a vacuum water bath, yielding the nonionic CO_2-WPU.

Cationic CO_2-WPU: Under a nitrogen atmosphere, 6 g of CO_2-polyol was charged into a 250-mL three-necked round-bottom flask equipped with a mechanical stirrer, a nitrogen inlet, and a condenser. The flask was heated in an oil bath to 70 °C. At this temperature, 2 g of IPDI and 5 mg of DBTDL were added. Once all hydroxyl groups in the reaction medium were consumed, 0.5 g of N-MDEA was introduced. Subsequently, an additional 1 g of IPDI was added after the NCO groups were completely reacted. Next, 0.15 g of TMP and 0.15 g of EDO were added sequentially, followed by a final addition of 0.1 g of EDO to achieve a high molecular weight polyurethane. THF was added during the reaction to reduce the viscosity of the mixture. To neutralize the system, 0.29 g of HCl (37%) was added, and 60 g of deionized water was gradually introduced dropwise under vigorous stirring to obtain the neutralized PU dispersion. The mixture was stirred for approximately 3 hours, and the THF was subsequently removed by rotary evaporation at 50 °C in a vacuum water bath, yielding the cationic CO_2-WPU.

3. Methods

Equipment

The ^{1}H nuclear magnetic resonance (NMR) spectra were recorded using a Bruker AVIII 400 MHz NMR spectrometer equipped with a Dual Probe, with chemical shifts (δ) reported in ppm relative to TMS and calibrated using residual solvent signals (DMSO-d_6: δ H = 2.5 ppm). Photoluminescence (PL) spectra were obtained using a Horiba Fluorolog-3 spectrofluorometer, while the absolute fluorescence quantum yield was determined with a calibrated Labsphere integrating sphere. Grayscale analysis was performed using ImageJ software, and all digital images were captured with a Canon EOS 7D camera. The particle size distribution was measured via dynamic light scattering (DLS) using a Malvern Zetasizer Nano ZS at room temperature. Water contact angles were measured with a Biolin Theta contact angle meter using a droplet volume of 4 μL. Scanning electron microscopy (SEM) images were acquired with a JEOL-6390 field emission scanning electron microscope, while surface morphology was characterized using a Bruker PT Scanning Probe Microscope (Dimension ICON). Weight monitoring was conducted with a KERN ABT 220-5DNM analytical balance. Fluorescent imaging was performed using an Upright Biological Microscope Ni-U with excitation at 330–385 nm UV light or a Zeiss Elyra-7 SIM system with 405 nm UV light.

Fluorescence microscopic imaging

Differential FM imaging. To prepare the AIEgen solution-1, 0.1 g of TPE-4S-Na was dissolved in 10 mL of deionized water. For the polymer emulsion sample, 0.01 g of CO_2-WPU (38 wt%) was diluted to a concentration of 0.05 wt%. Then, 0.019 mL of AIEgen solution-1 was added to the diluted CO_2-WPU (5 wt% in solid), giving the polymer emulsion sample. 0.1g of inorganic pigment or filler was added to 5 mL of water under stirring, the mixture was then diluted to a concentration of 0.05 wt%, and 0.019 mL of AIEgen solution-1 was subsequently added, leading to the formation of the inorganic emulsion sample. All of the obtained emulsion samples were spin-coated onto glass slides and then subjected to FM imaging under an upright biological microscope (Ni-U) with 330-385 nm UV light as the exciting light source, or under Zeiss Elyra-7 SIM mode with 405 nm UV light.

Application in the coatings industry. A TD emulsion was prepared by dispersing 5 g of TD powder in 20 mL of water under stirring. The CC and TP emulsion was prepared with the same procedure. Next, 3 g of the TD emulsion was added to 10 g of CO_2-WPU under stirring, and 3 g of the CC and TP emulsions were further added. Then, 0.1 g of blended emulsion was diluted to a concentration of 0.2 wt% using water, and 0.5 mL of AIEgen solution-1 was added for final fluorescence imaging.

Monitoring of film formation

At the molecular level. To prepare the AIEgen solution-2, 0.5g of TPE-4S-Na was dissolved in 5 mL of deionized water. For the monitoring sample, 0.19 mL of the AIEgen solution was added to 1 g of CO_2-WPU. The resulting mixture was used for monitoring purposes. To initiate the monitoring process, the sample was dropped onto a quartz plate. The drying process of the wet film was immediately monitored using dynamic PL measurements at 448 nm with excitation of 365 nm UV light. To prevent possible photobleaching caused by continuous exposure to UV light during the monitoring period, the excitation light was intermittently shielded, resulting in discontinuous signals.

At the microscopic level. 0.1 g of CO_2-WPU was diluted to a concentration of 5 wt% using water. Next, 0.038 mL of AIEgen solution-2 was added to the diluted CO_2-WPU. For the monitoring process, a small drop of the sample was coated onto a glass slide. The wet film was immediately subjected to FM to monitor the film-forming process. To observe different regions of the wet film in real-time, fixed-sized regions of 1.24 mm * 0.88 mm were selected at various positions, as illustrated in Figure 4e, f. Furthermore, the sample was further diluted to a concentration of 0.05 wt% and coated onto a glass slide. The coated slide was then dried at two different temperatures of 5°C and 25°C, respectively, and finally monitored under super-resolution FM, as shown in Figure 4a, b. The emulsion samples were diluted and drop-cast onto glass slides (25.4 × 76.2 mm, thickness: 1.0–1.2 mm), followed by drying at room temperature. The morphology of the emulsion particles was observed under a fluorescence microscope, and their sizes were measured using ImageJ software.

At the macroscopic level. 0.38 mL of AIEgen solution-2 was added to 1 g of CO_2-WPU. For the monitoring process, 0.2 mL of the sample was coated onto a glass slide and immediately subjected to the monitoring platform, as depicted in Figure 5a. The entire drying process was recorded using a camera under UV light with a wavelength of 365 nm. The captured images were then processed using ImageJ software to read the grayscale information.

Application in industry. To realize the practical application in the coatings industry, an industrialized wood coating was

selected as the study object. Similarly, 2.5 g of TPE-NS-Na was dissolved in 15 g of deionized water, and then added to 100 g of the industrialized waterborne wood coating (provided by Carpoly Chemical Group Co., Ltd.). Subsequently, the sample was spray-coated onto a primed wood board (30 cm * 20 cm). The coated board was immediately subjected to the monitoring platform, images, as well as the grayscale changes, and drying film formation were monitored using the previously described methods.

4. Results and discussion

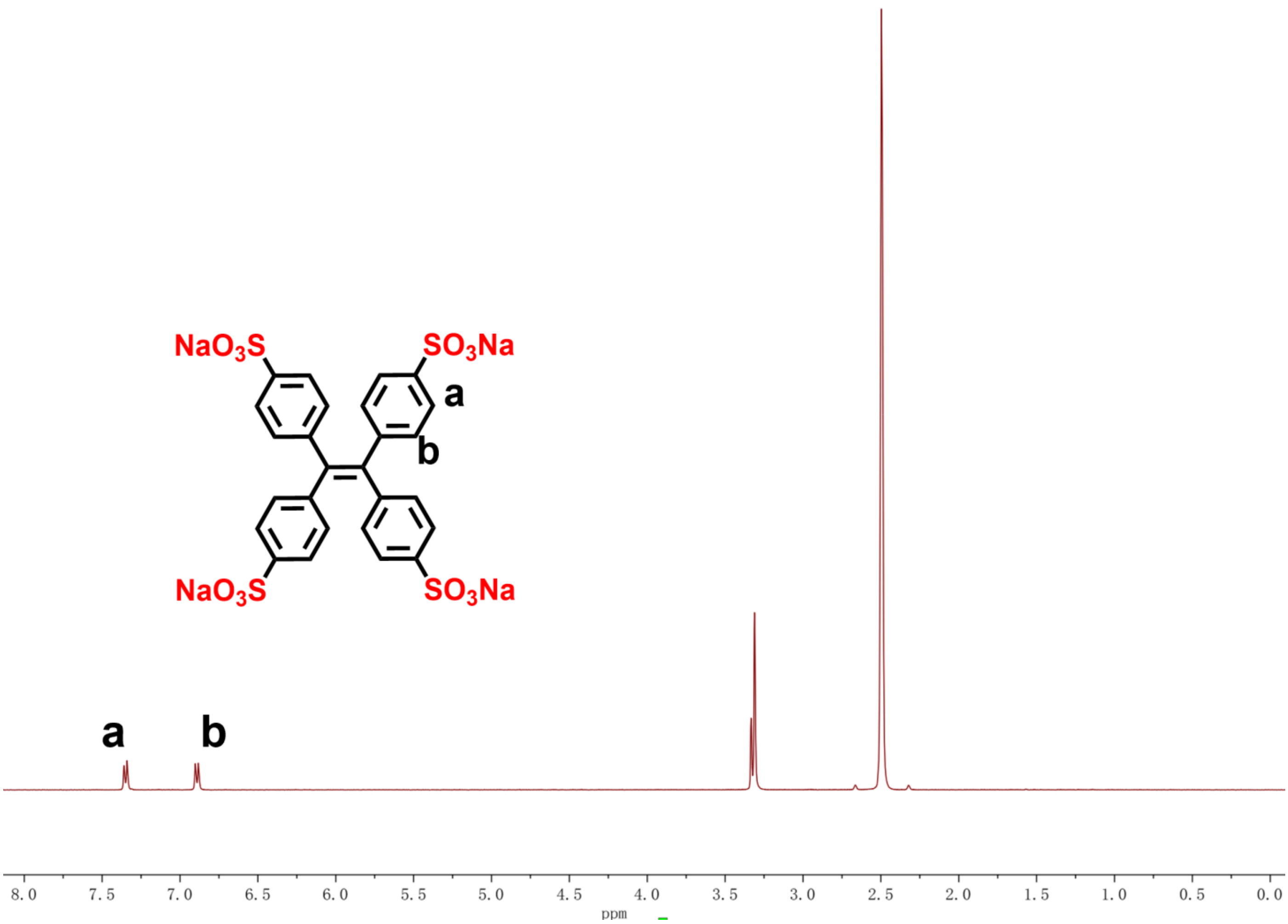

Figure S1 The ^{1}H-NMR spectrum of TPE-4S-Na in D-DMSO.

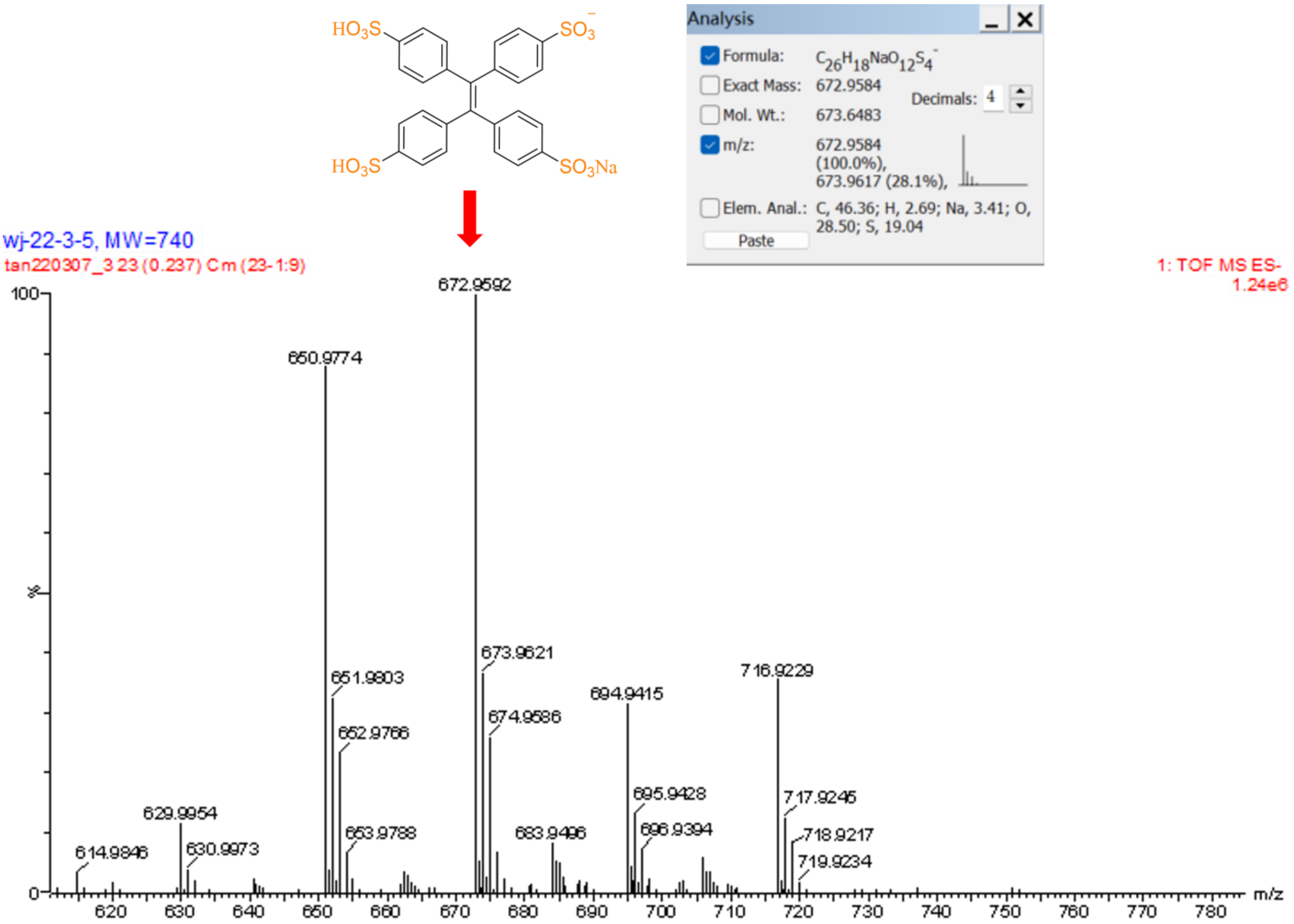

Figure S2 The HRMS spectrum of TPE-4S-Na.

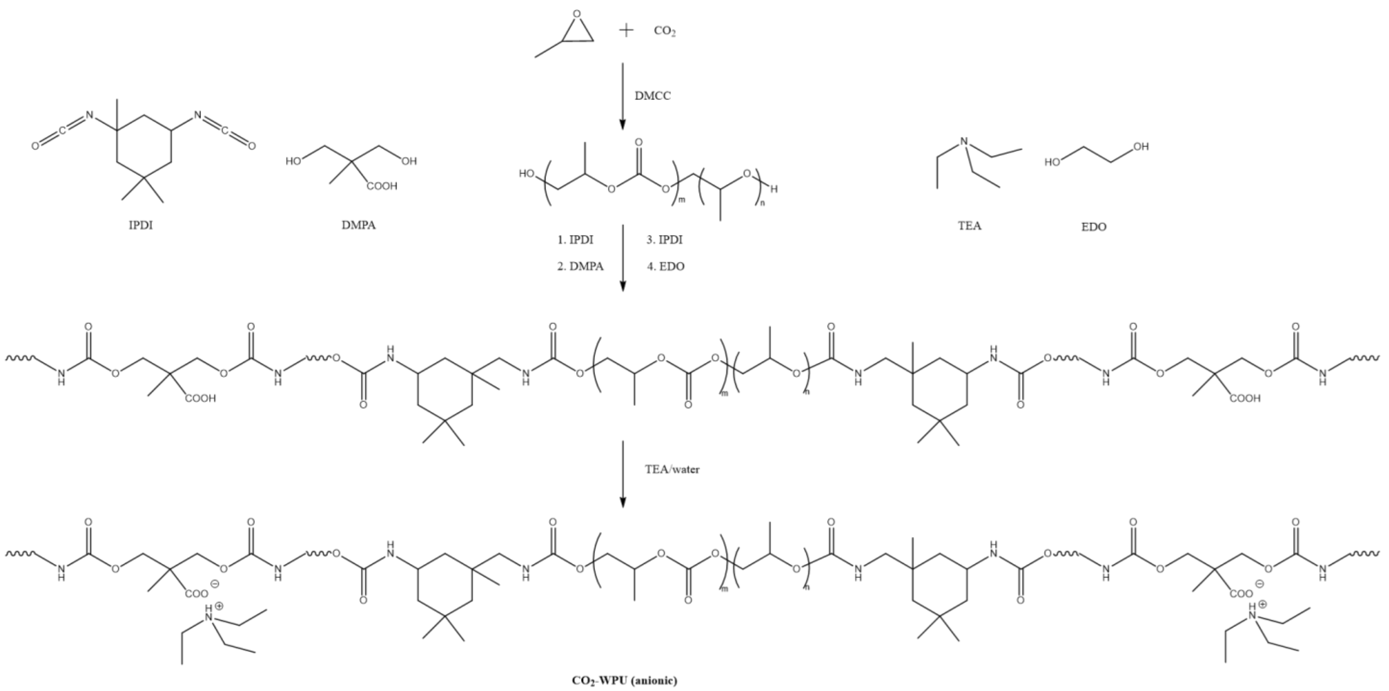

Scheme S1 The preparation procedure of CO_2-WPU (anionic).

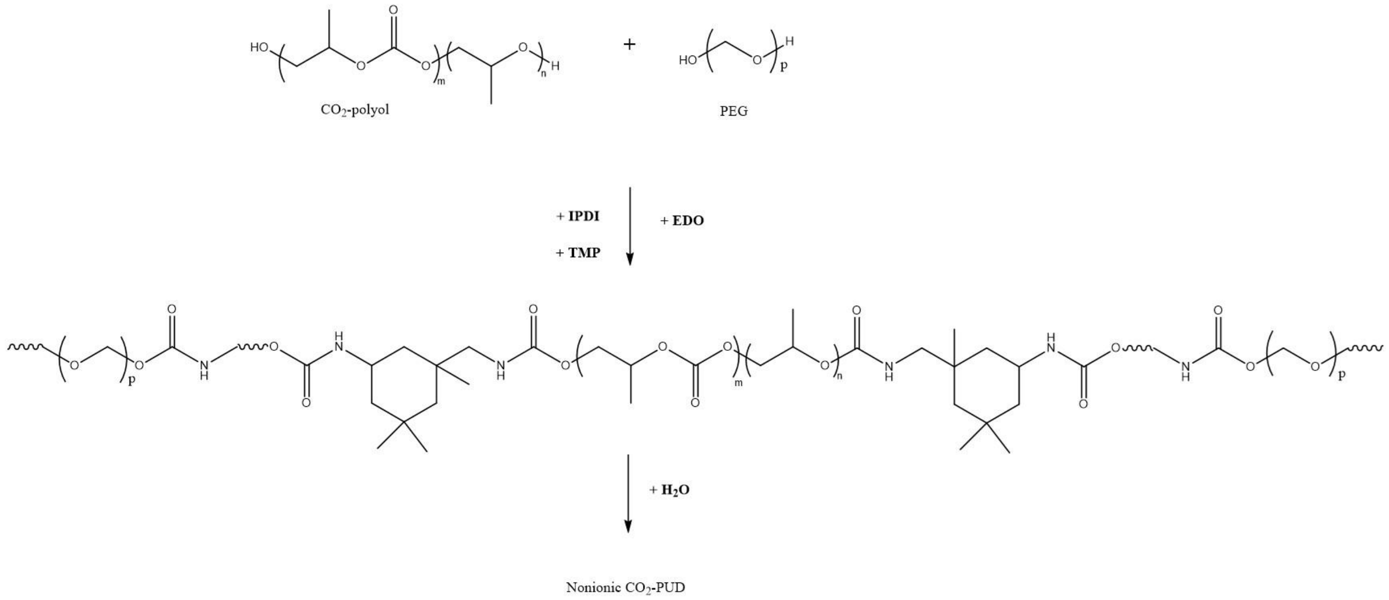

Scheme S2 The preparation procedure of nonionic CO_2-WPU.

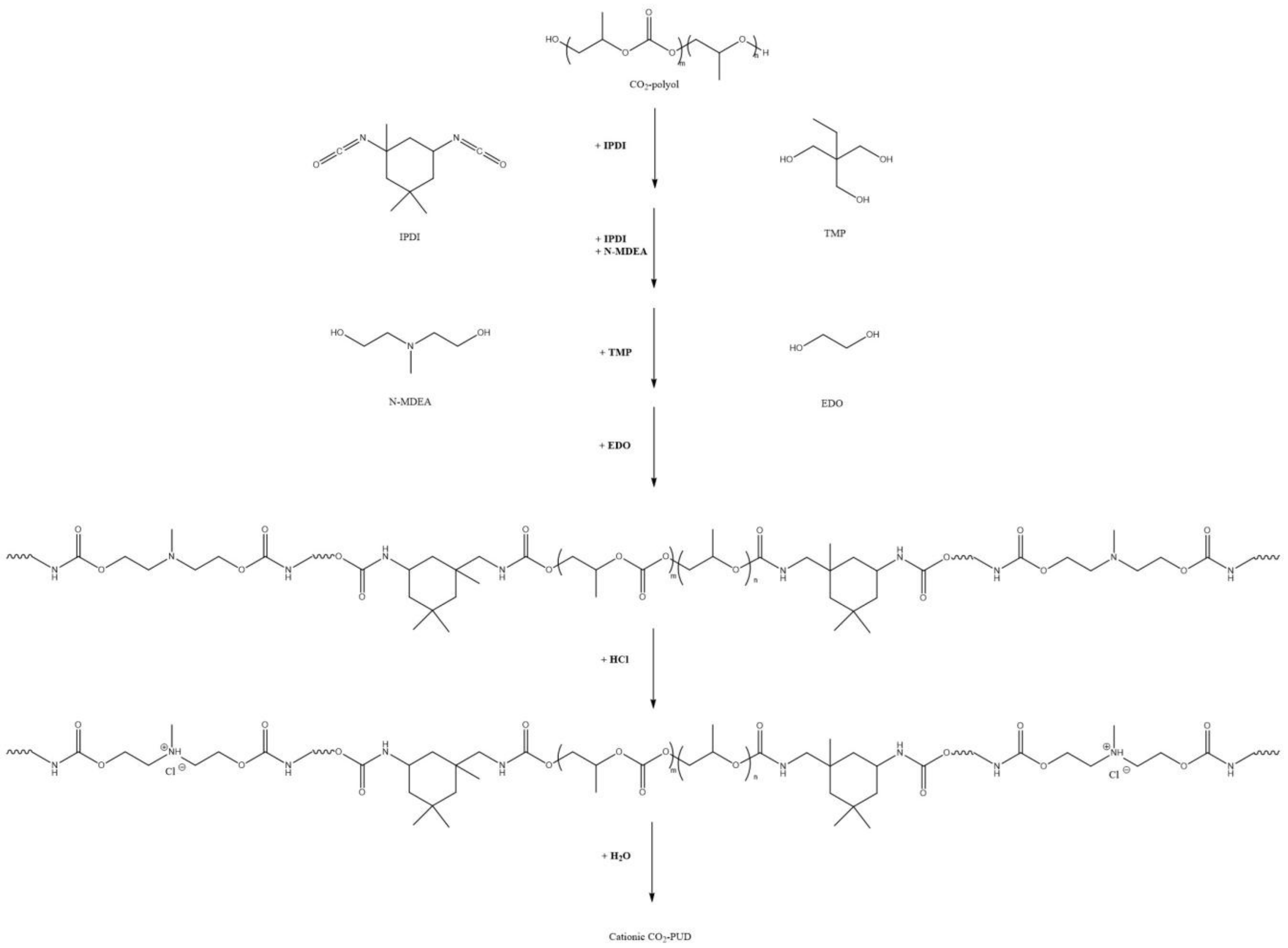

Scheme S3 The preparation procedure of cationic CO_2-WPU.

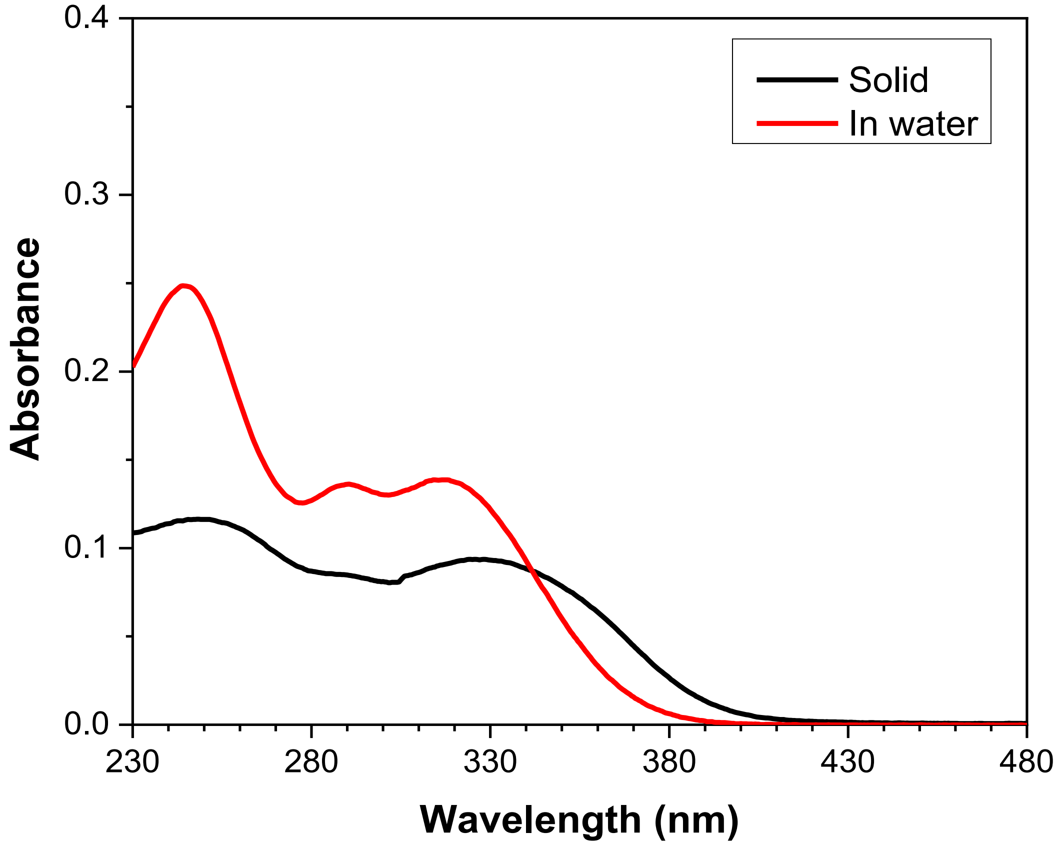

Figure S3 The Abs spectra of TPE-4S-Na in solid and water solution.

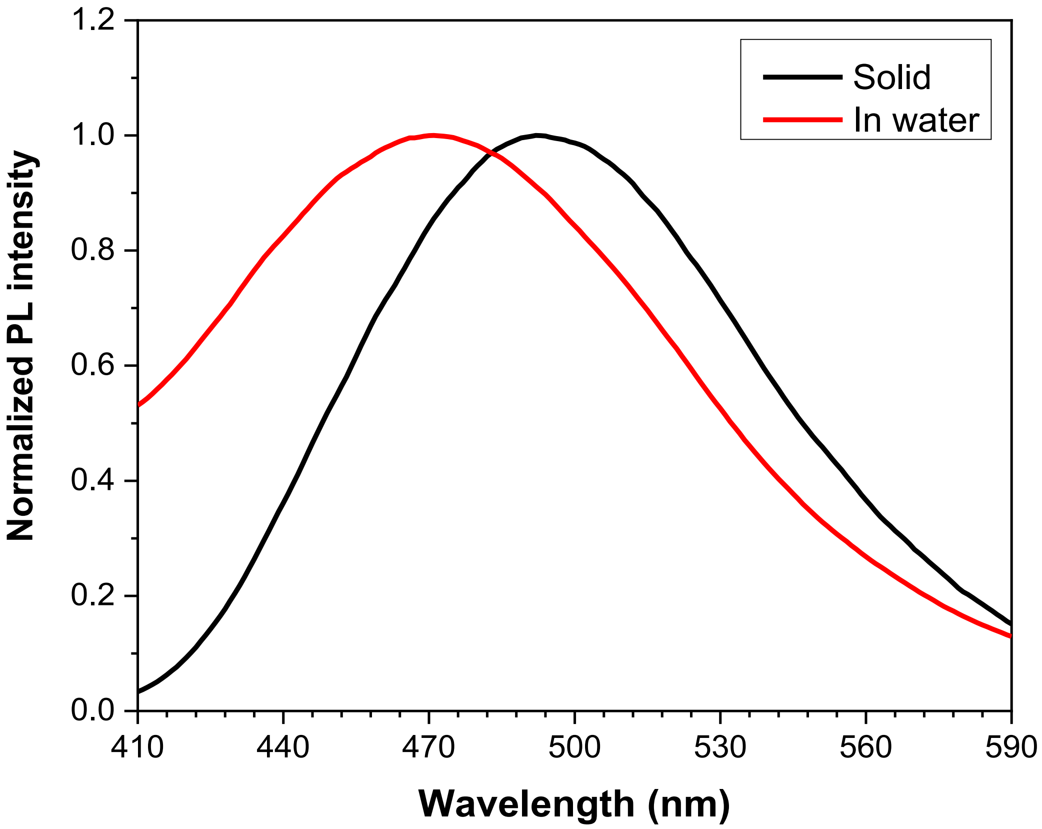

Figure S4 The PL spectra of TPE-4S-Na in solid and water solution.

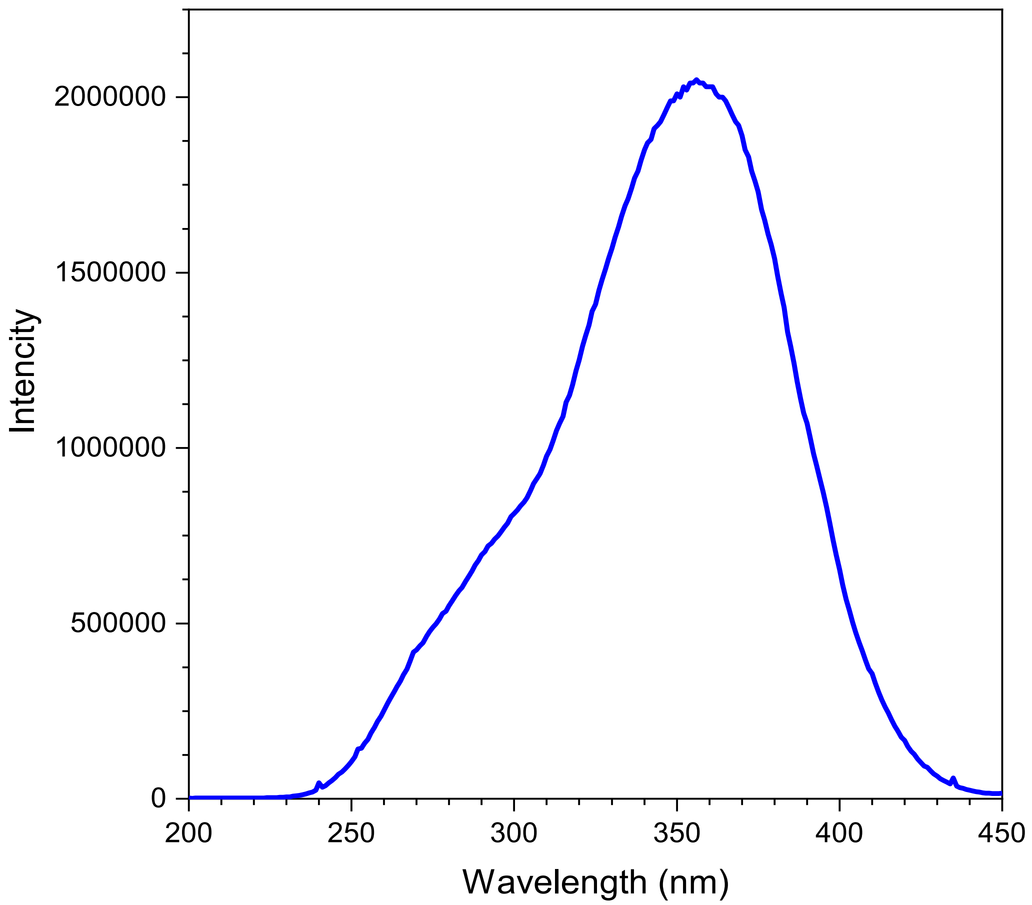

Figure S5 The excitation spectrum of TPE-4S-Na in water solution.

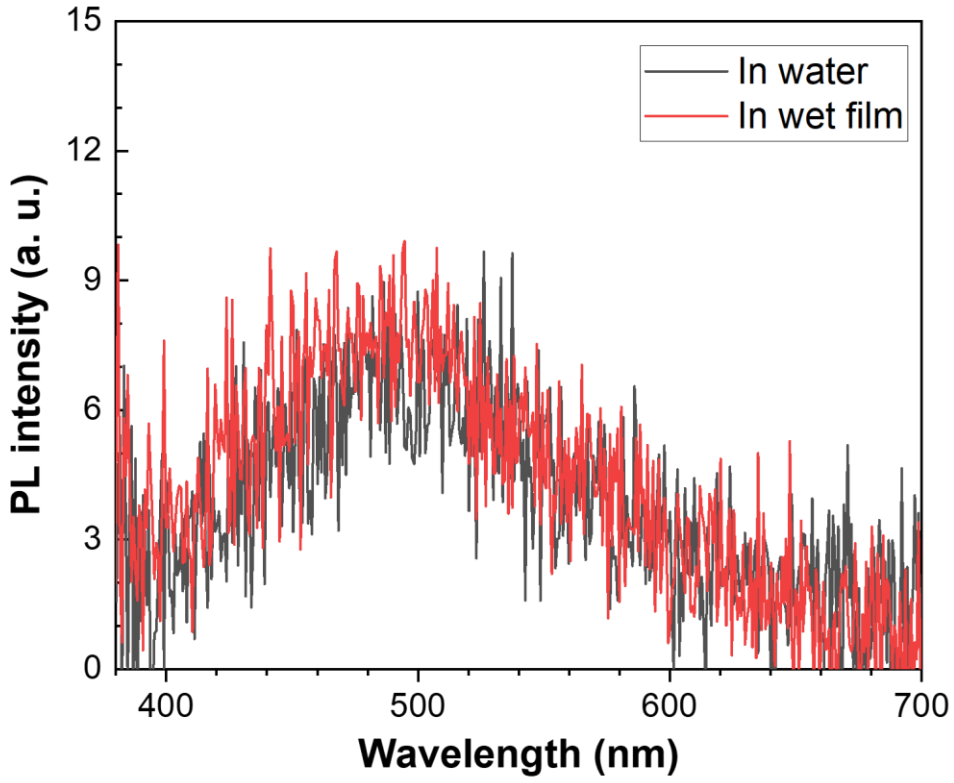

Figure S6 The PL spectrum of TPE-4S-Na in water and in wet film (according to QY test data).

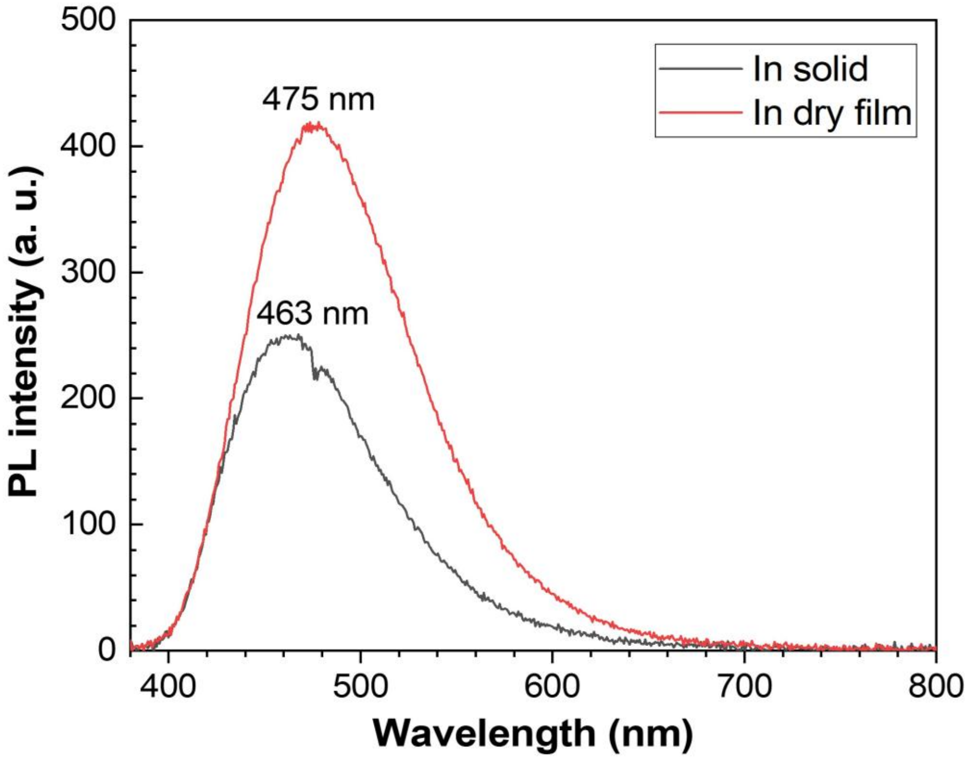

Figure S7 The PL spectrum of TPE-4S-Na in solid and in dry film (according to QY test data).

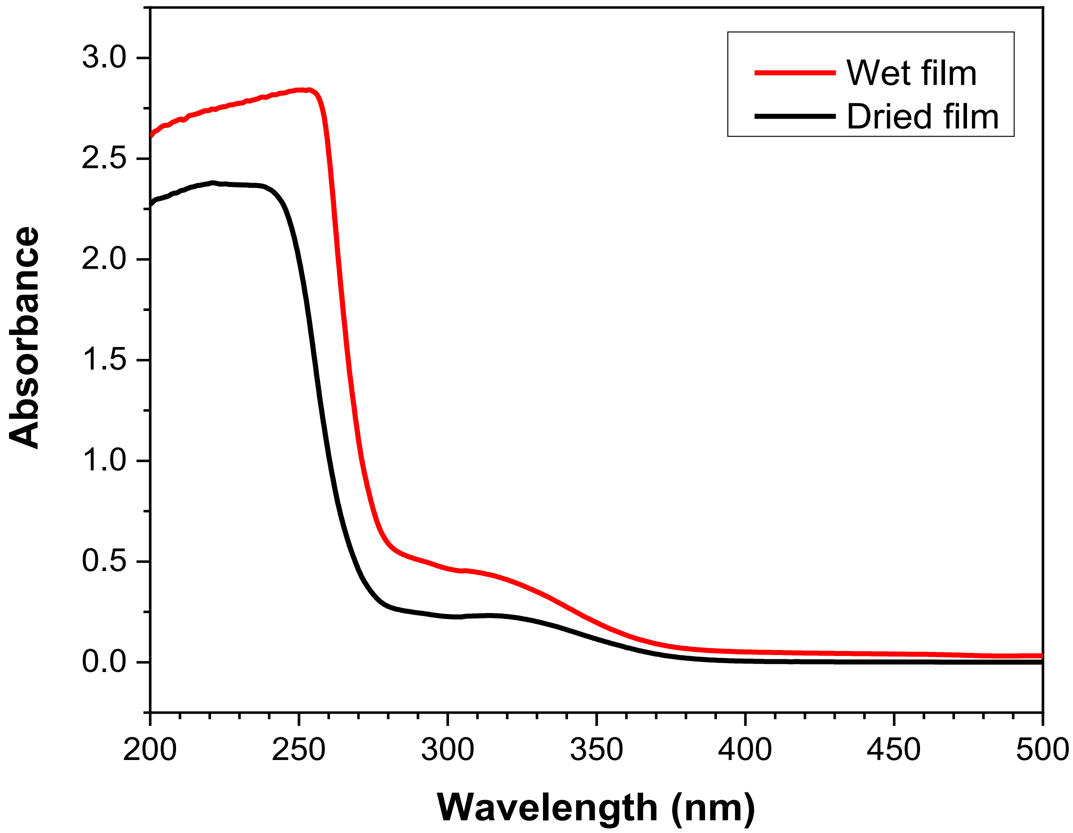

Figure S8 The UV-vis spectra of TPE-4S-Na blended polymer emulsion before and after film formation.

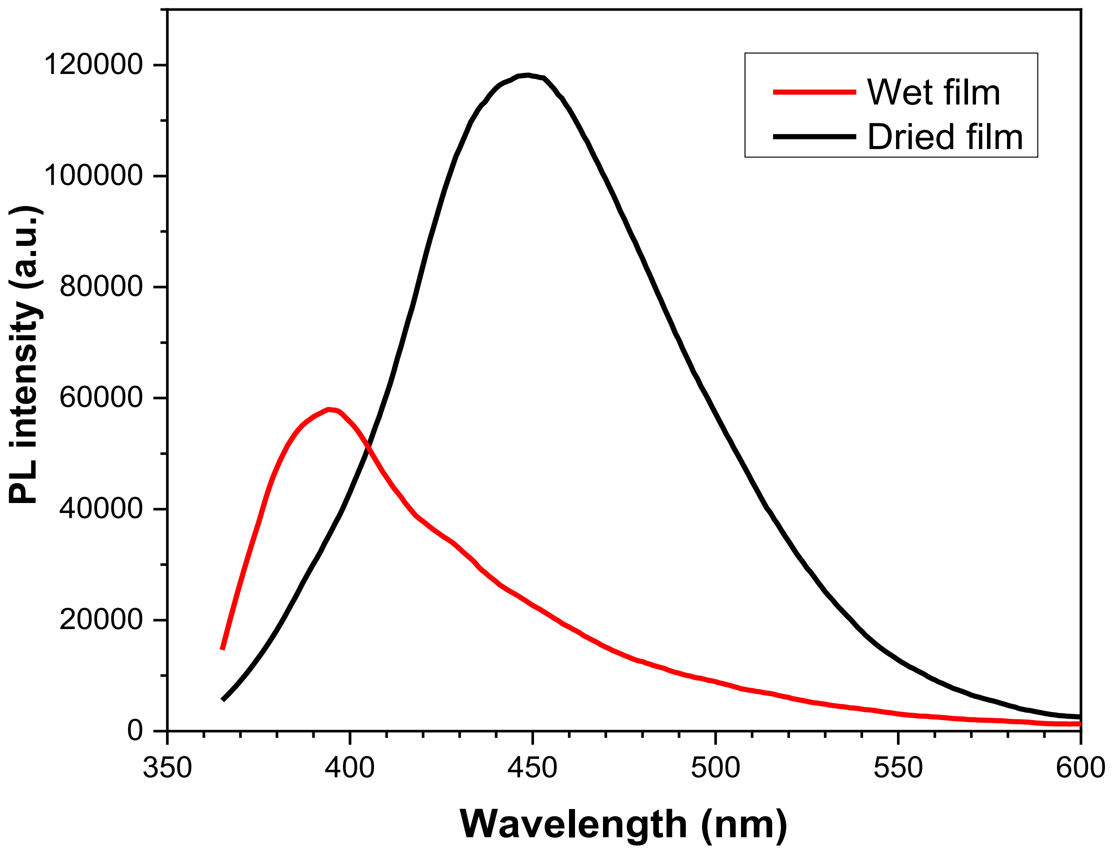

Figure S9 PL spectra of TPE-4S-Na blended polymer emulsion before and after film formation.

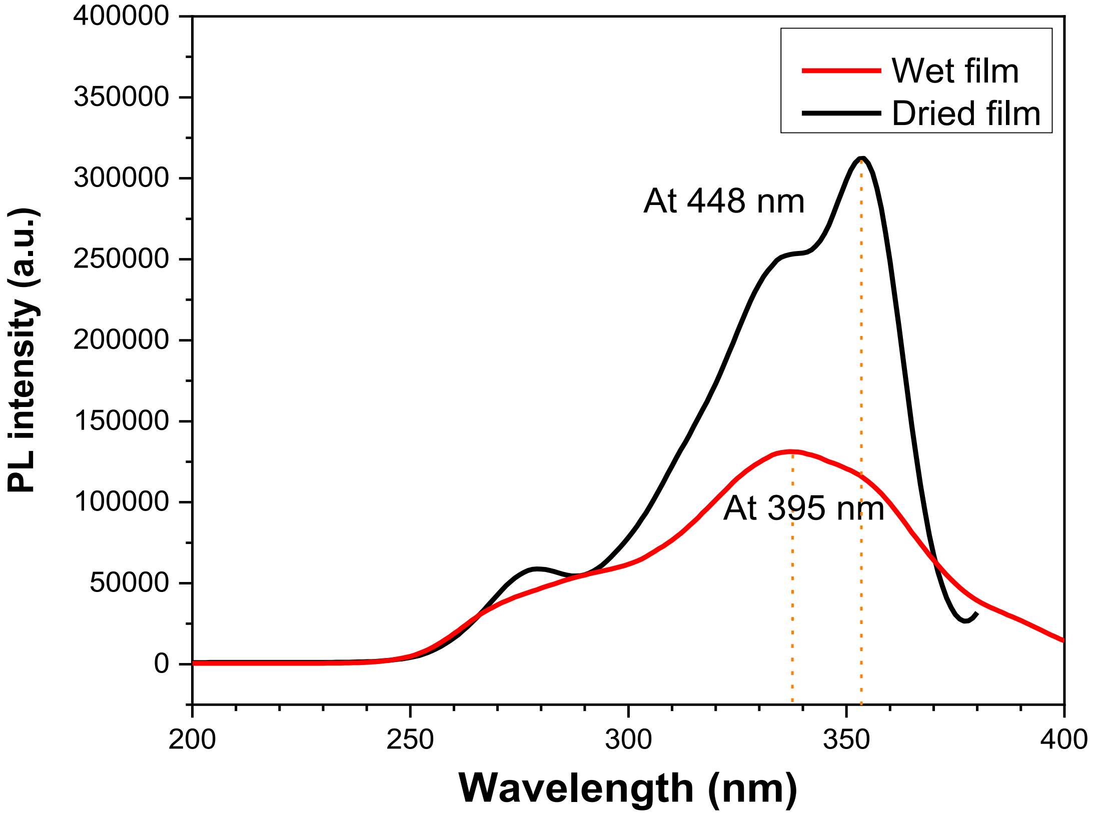

Figure S10 The excitation spectra of TPE-4S-Na blended polymer emulsion before and after film formation.

Discussion S1: As shown in **Figure S9**, the dried film containing TPE-4S-Na showed a PL peak at around 448 nm; thus, the dynamic PL measurements were set at 448 nm. As shown in **Figure S10**, the wet and dried films showed similar excitation spectra with PL peaks set at 395 nm and 448 nm, respectively. Considering 365 nm is the ordinarily used excitation light source, thus, the excitation wavelength was set as 365 nm in this research.

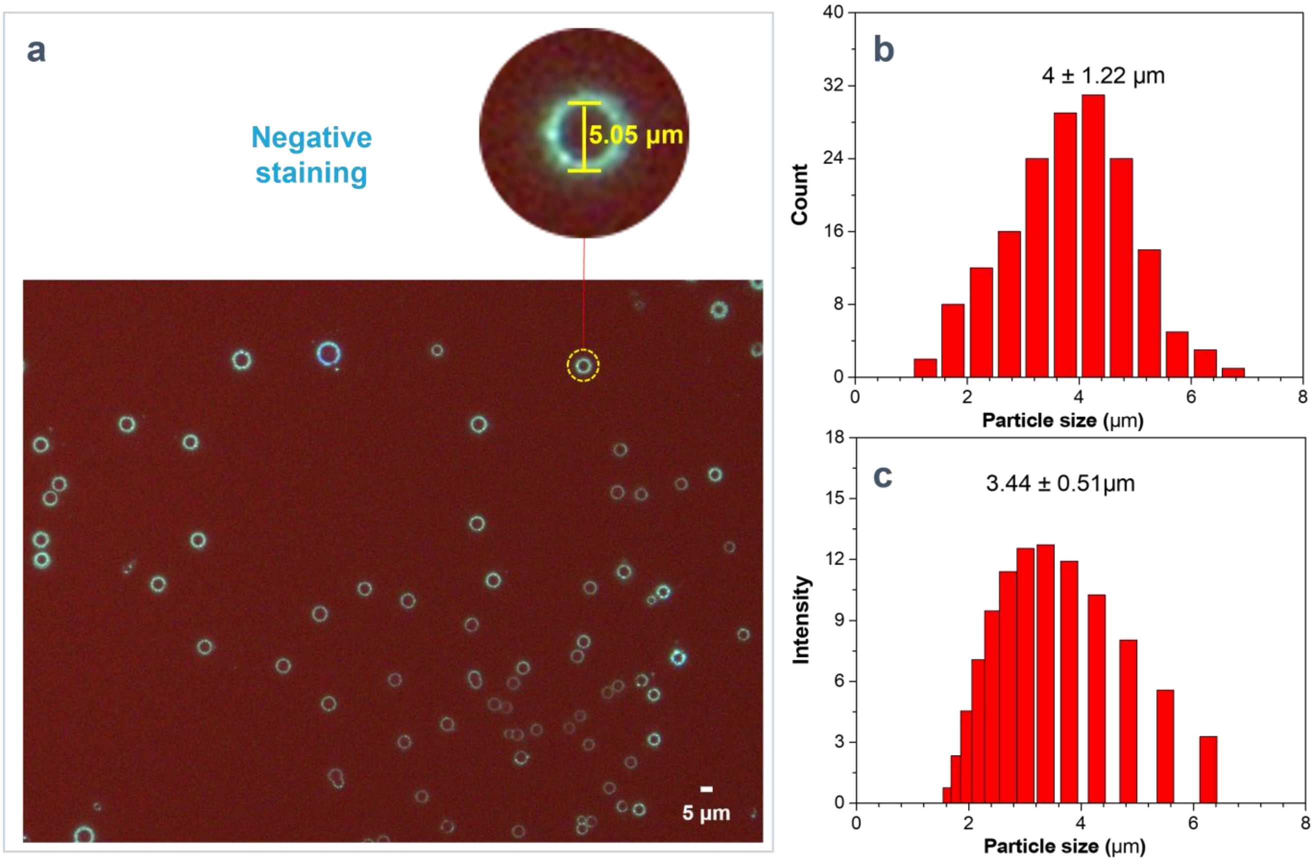

Figure S11 a) Fluorescence microscopy image for CO_2-WPU with particle size to be 4 μm; b) The column of particle size distribution calculated from fluorescence microscopy image; c) The column of particle size distribution based on DLS test.

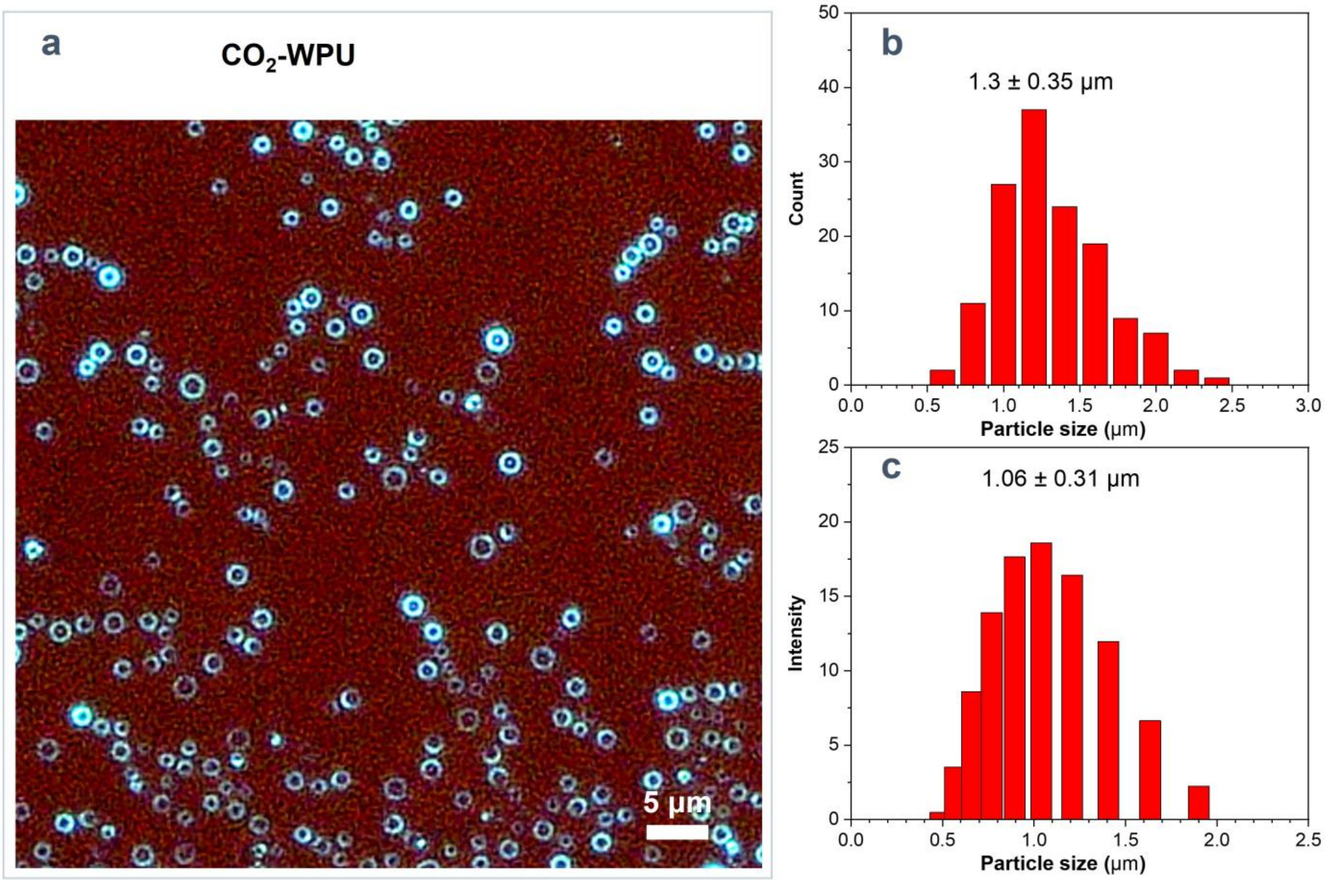

Figure S12 a) Fluorescence microscopy image for CO_2-WPU with particle size to be 1.3 μm; b) The column of particle size distribution calculated from fluorescence microscopy image; c) The column of particle size distribution based on DLS test.

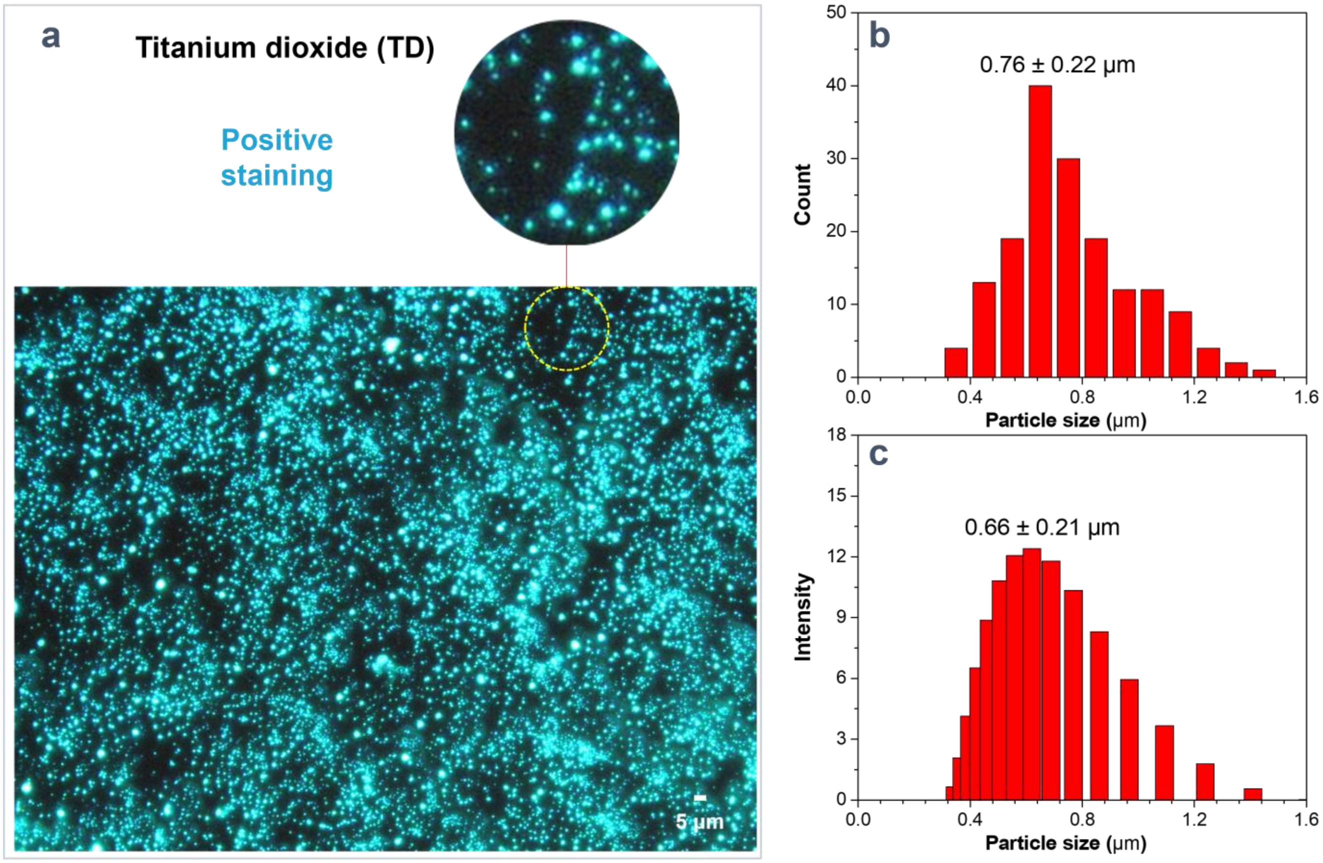

Figure S13 a) Fluorescence microscopy image for titanium dioxide; b) The column of particle size distribution calculated from fluorescence microscopy image; c) The column of particle size distribution based on DLS test.

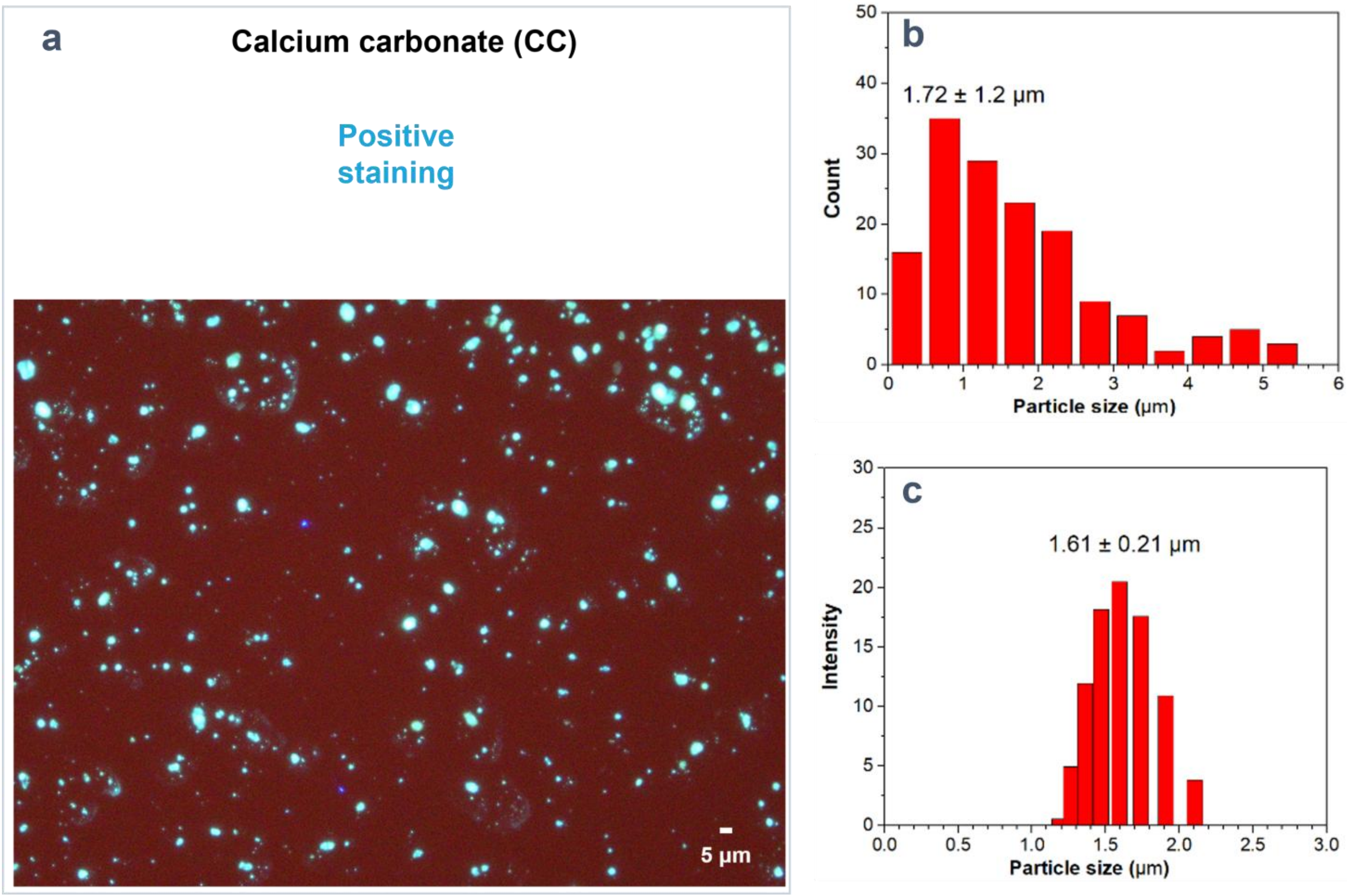

Figure S14 a) Fluorescence microscopy image for calcium carbonate; b) The column of particle size distribution calculated from fluorescence microscopy image; c) The column of particle size distribution based on DLS test.

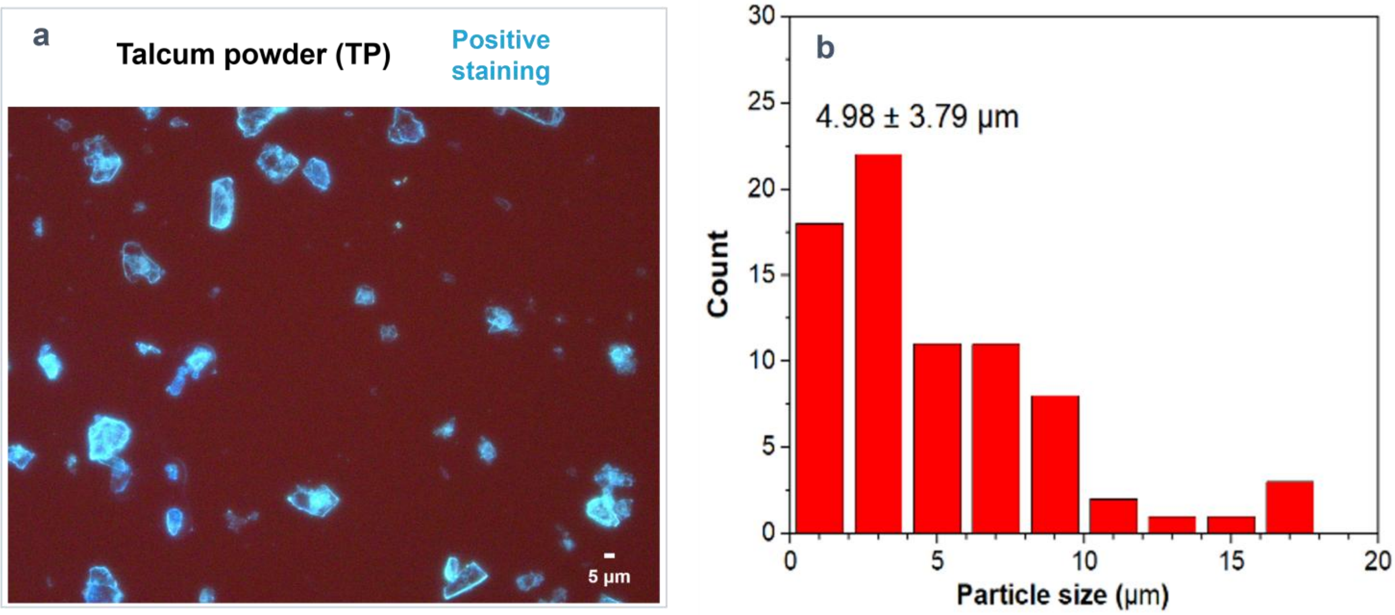

Figure S15 a) Fluorescence microscopy image of talcum powder; b) The column of particle size distribution calculated from fluorescence microscopy image; c) The column of particle size distribution based on DLS test.

Discussion S2: As shown in **Figure S11-15**, the polymer emulsion showed a negative staining effect, while most frequently used inorganic pigments or fillers in the coatings industry, like particles of titanium dioxide, calcium carbonate, and talcum powder, showed a positive staining effect. The particle size read from fluorescence microscopic images was similar to that of the DLS test, showing reliability in particle size measurement under FM imaging.

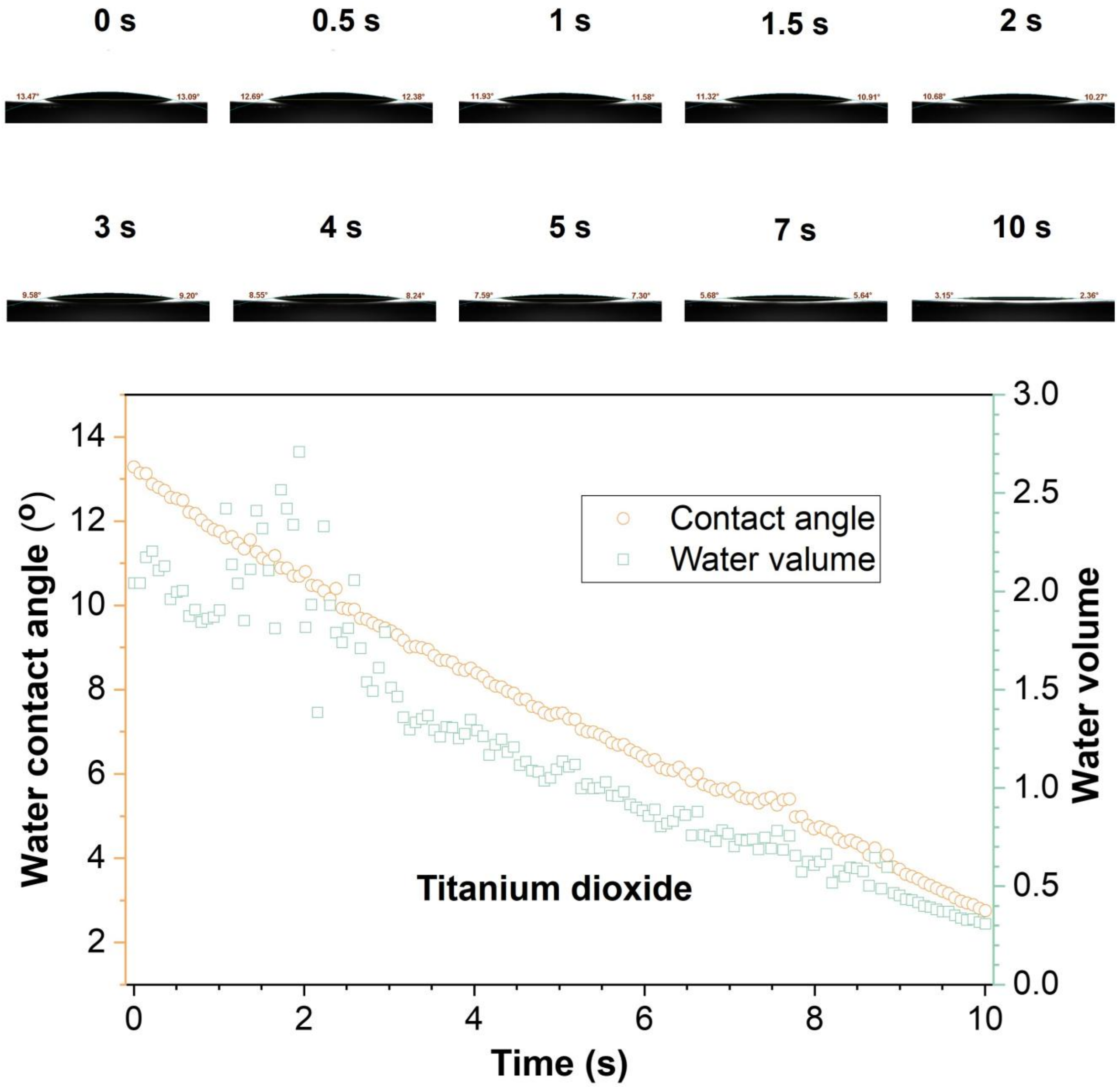

Figure S16 The water contact angle and water volume versus time for titanium dioxide prepared with tablet pressing (2 tons pressure), as well as contact angle image at different times.

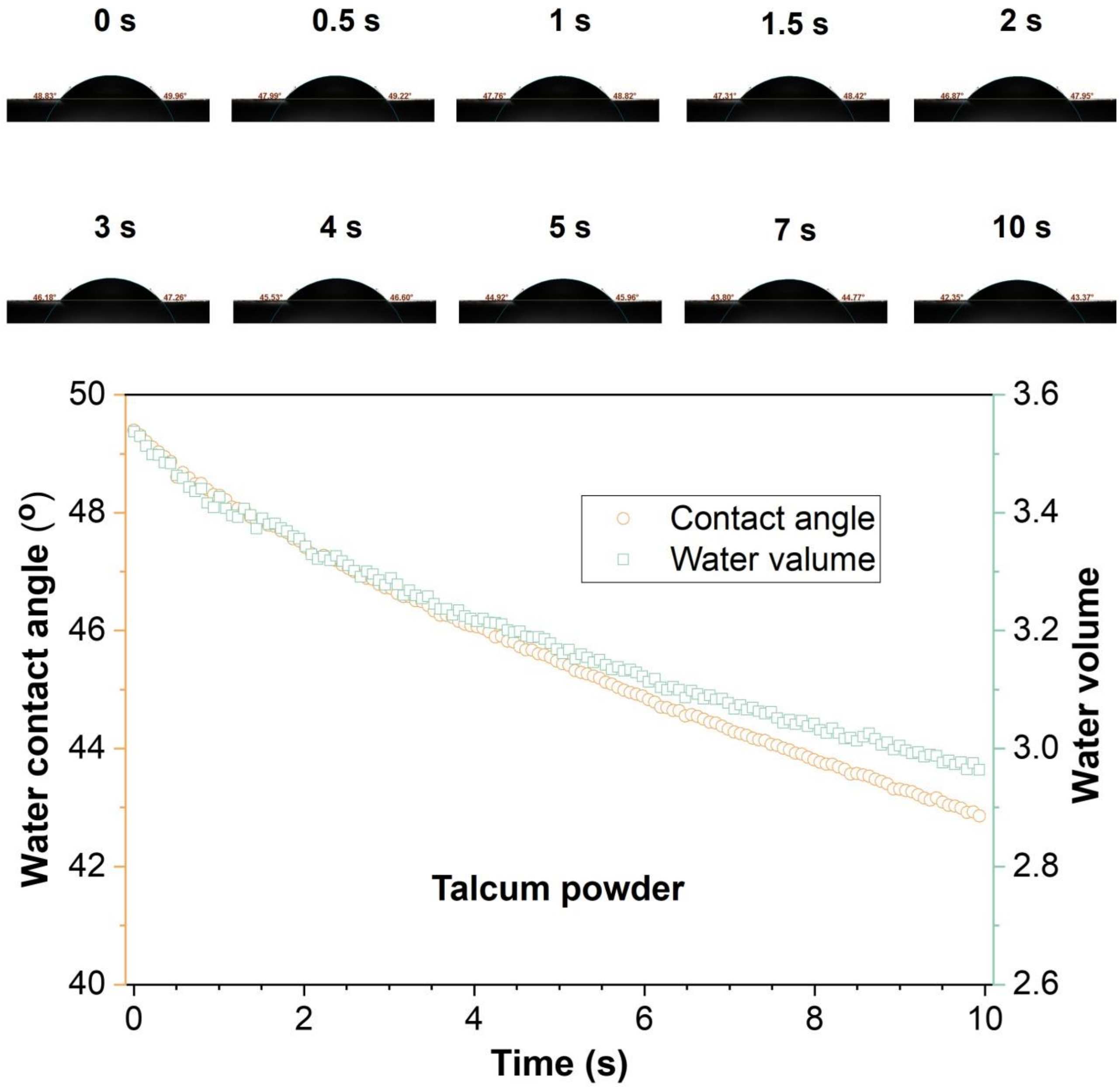

Figure S17 The water contact angle and water volume versus time for talcum powder prepared with tablet pressing (2 tons pressure), as well as contact angle image at different times.

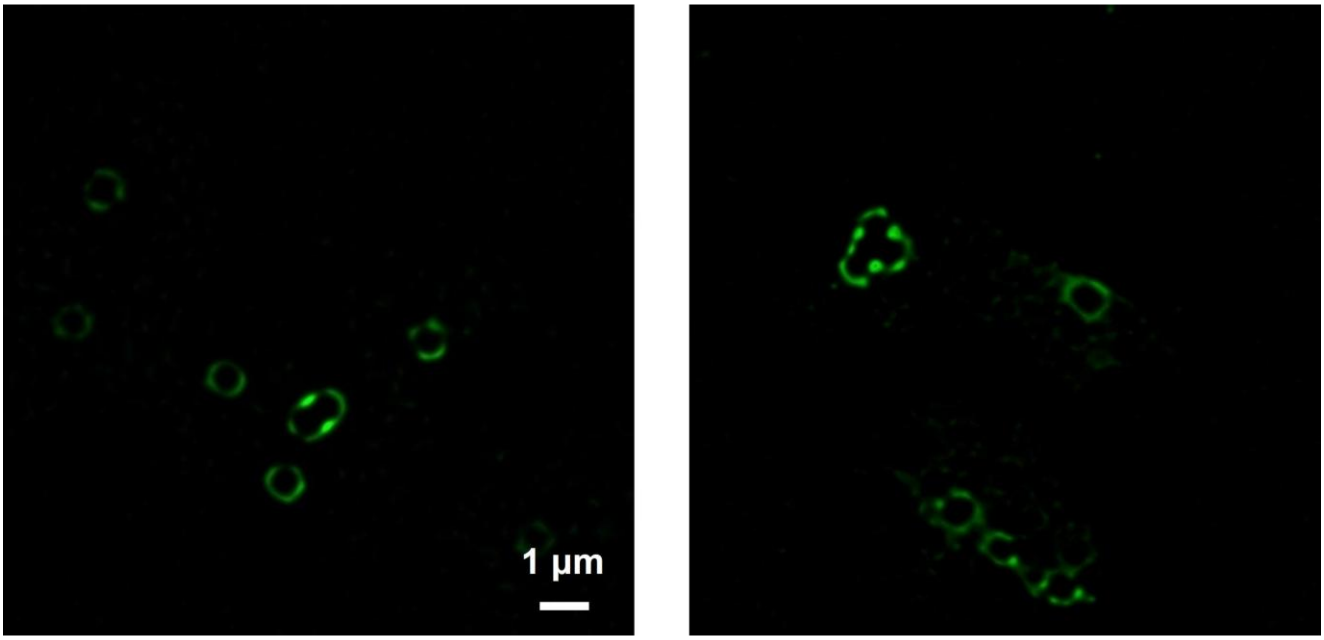

Figure S18 a) Super-resolution FM images of fused CO_2-WPU particles (average particle size: 680 nm) with particle numbers to be two and three at 25 °C, respectively.

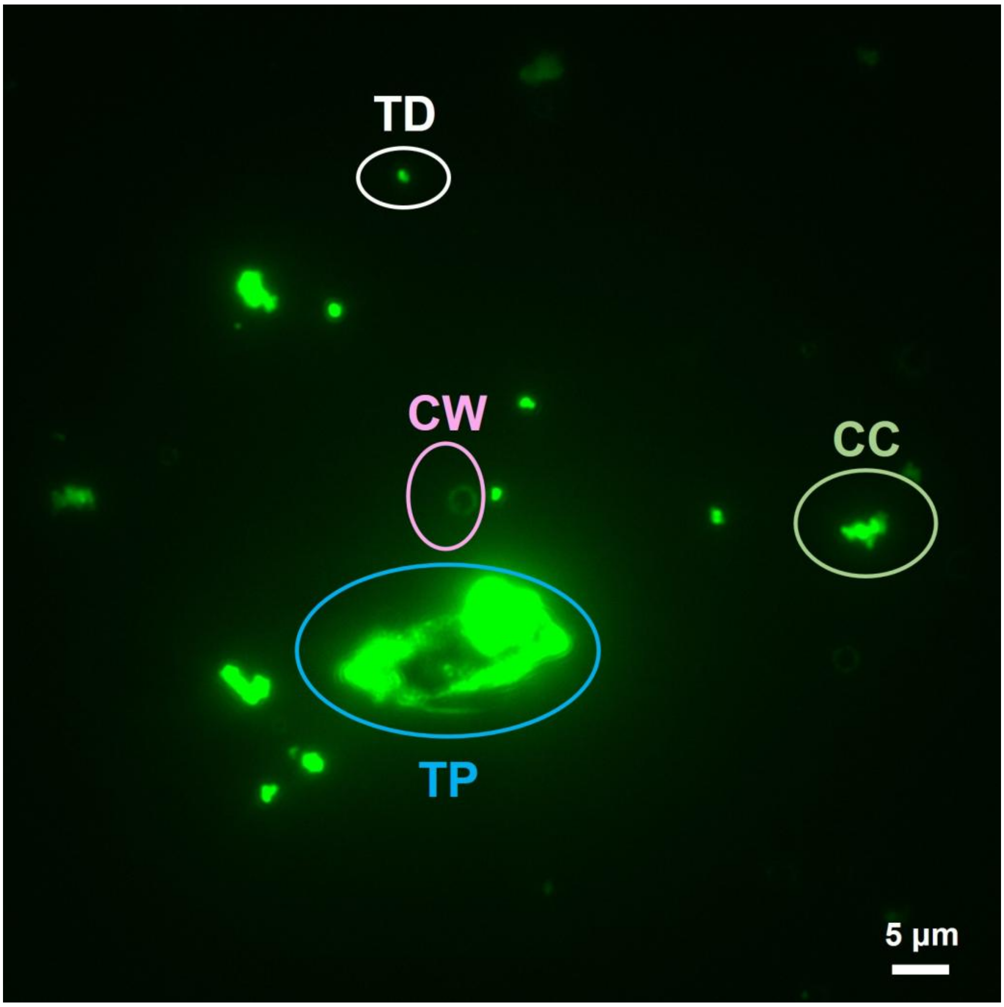

Figure S19 The FM images of CO_2-WPU particles after blending with TD, CC, and TP. All polymer particles and inorganic particles were diluted to 0.05 wt%, with 5 wt% TPE-4S-Na as a fluorescent probe in the solid content. Scale bar: 5 μm.

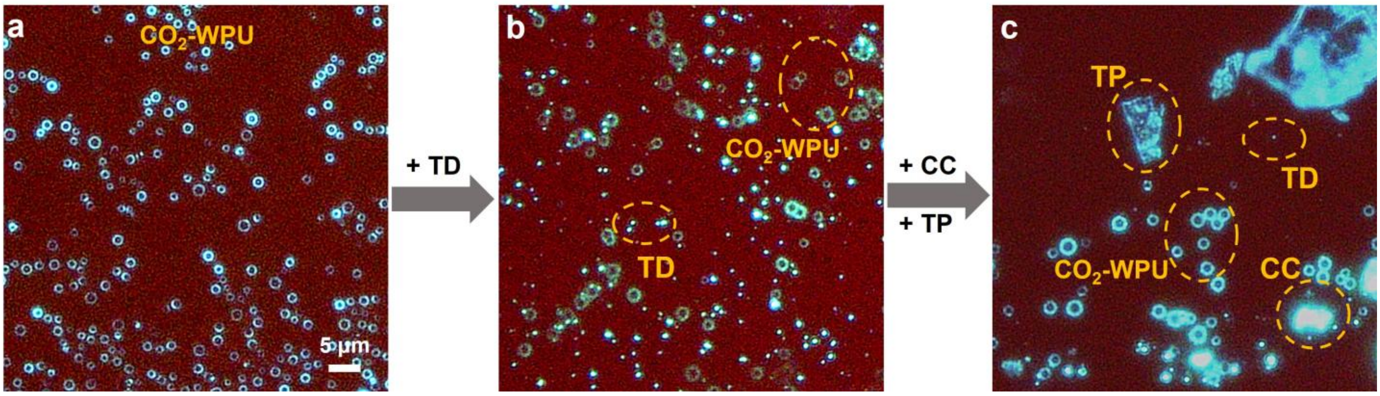

Figure S20 The FM images of CO_2-WPU particles (average particle size: 1.3 μm) before and after blending with TD, as well as further blending with CC and TP, respectively. All polymer particles and inorganic particles were diluted to 0.05 wt%, with 5 wt% TPE-4S-Na as a fluorescent probe in the solid content. Scale bar: 5 μm.

Discussion S3: As shown in **Figure S20a**, TPE-4S-Na displayed negative staining to CO_2-WPU particles. When inorganic particles of titanium dioxide (TD) were blended into the polymer emulsion under stirring, both negatively stained polymer particles and positively stained TD particles were observed in **Figure S20b**. Further addition of CC and talcum powder (TP) resulted in the appearance of these newly added positively stained inorganic particles (**Figure S20c**). Thanks to the differential staining characteristics of TPE-4S-Na for organic and inorganic particles, as well as its simple operational procedures and low cost, these practical results demonstrate the excellent application prospects of AIEgens for fluorescence imaging in the waterborne coatings industry.

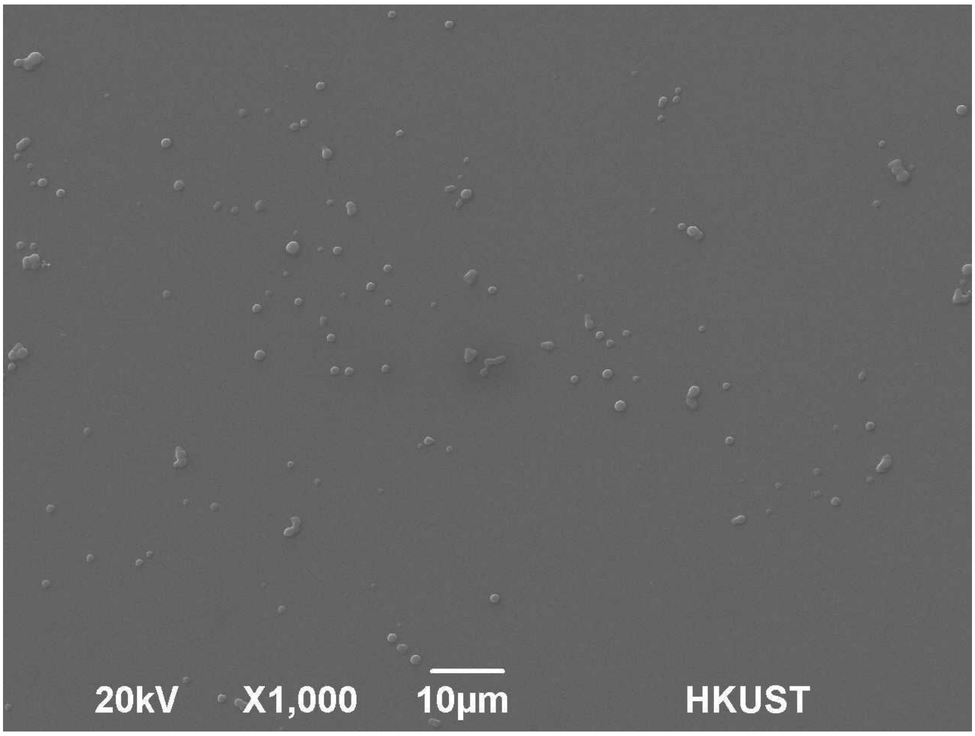

Figure S21 The SEM image of CO_2-WPU.

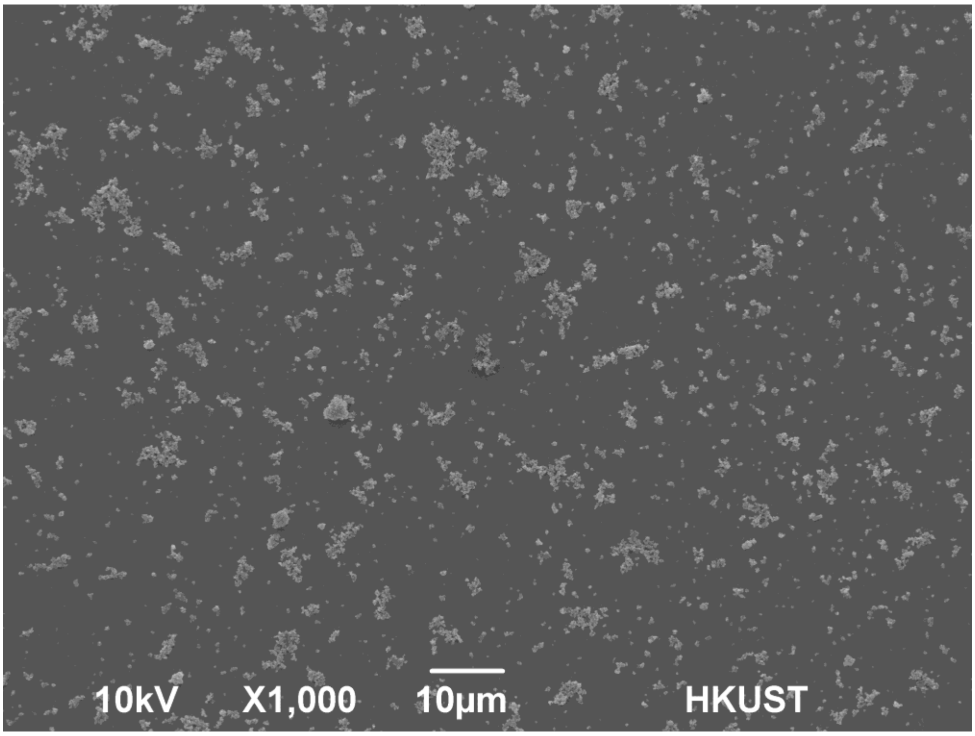

Figure S22 The SEM image of titanium dioxide.

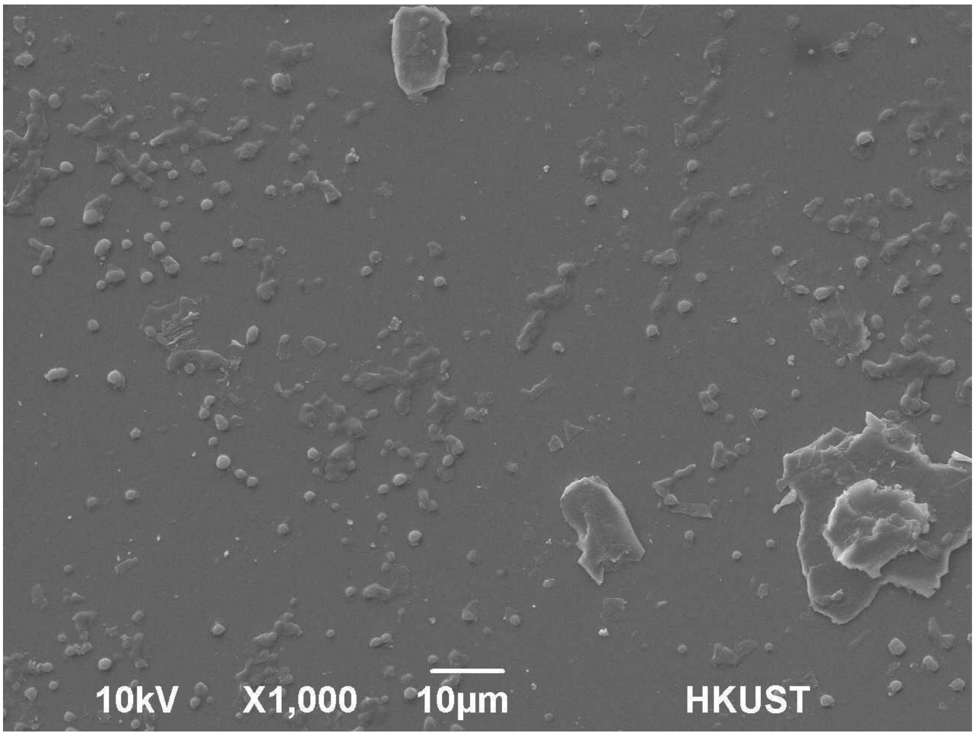

Figure S23 The SEM image of blending system.

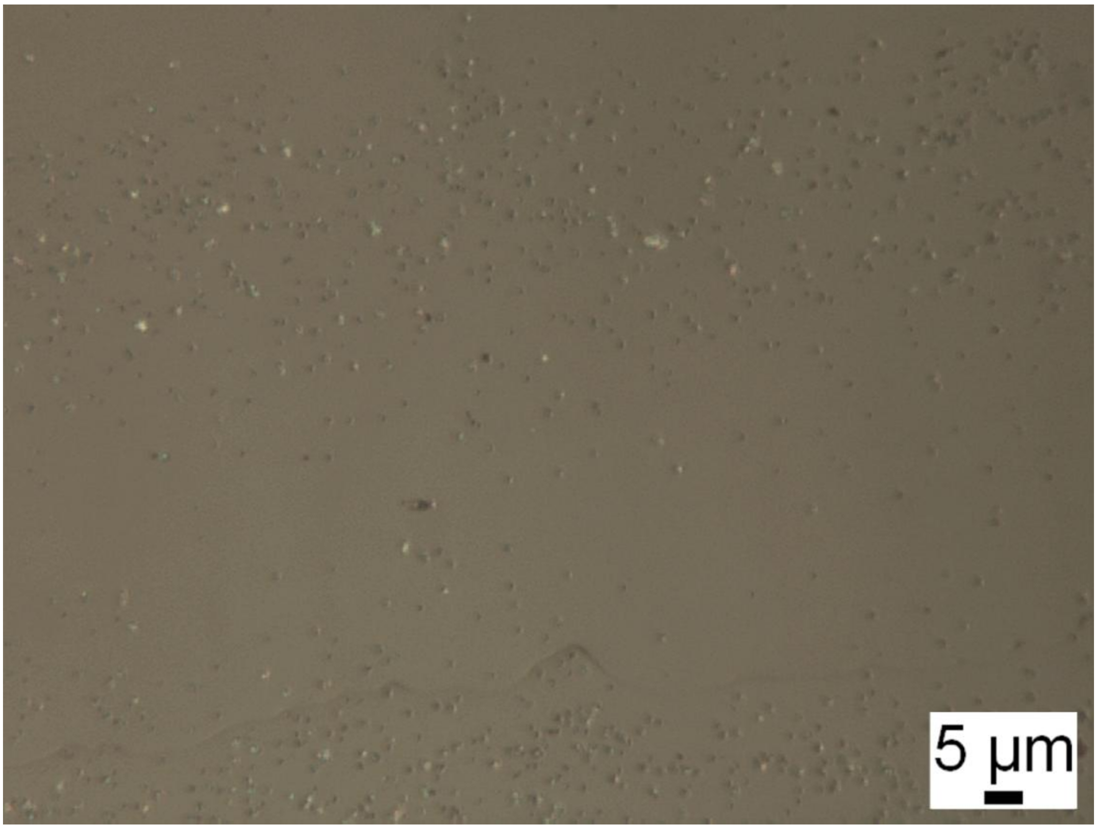

Figure S24 The optical microscopy image of titanium dioxide.

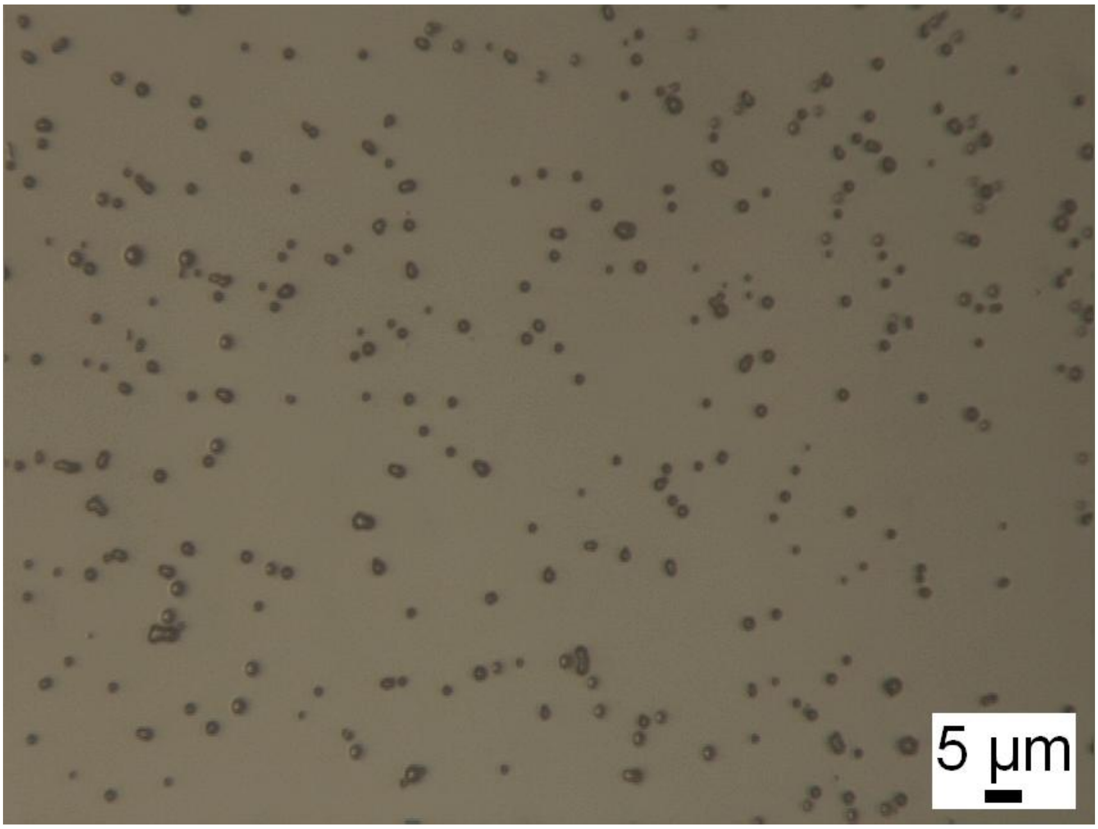

Figure S25 The optical microscopy image of CO_2-WPU.

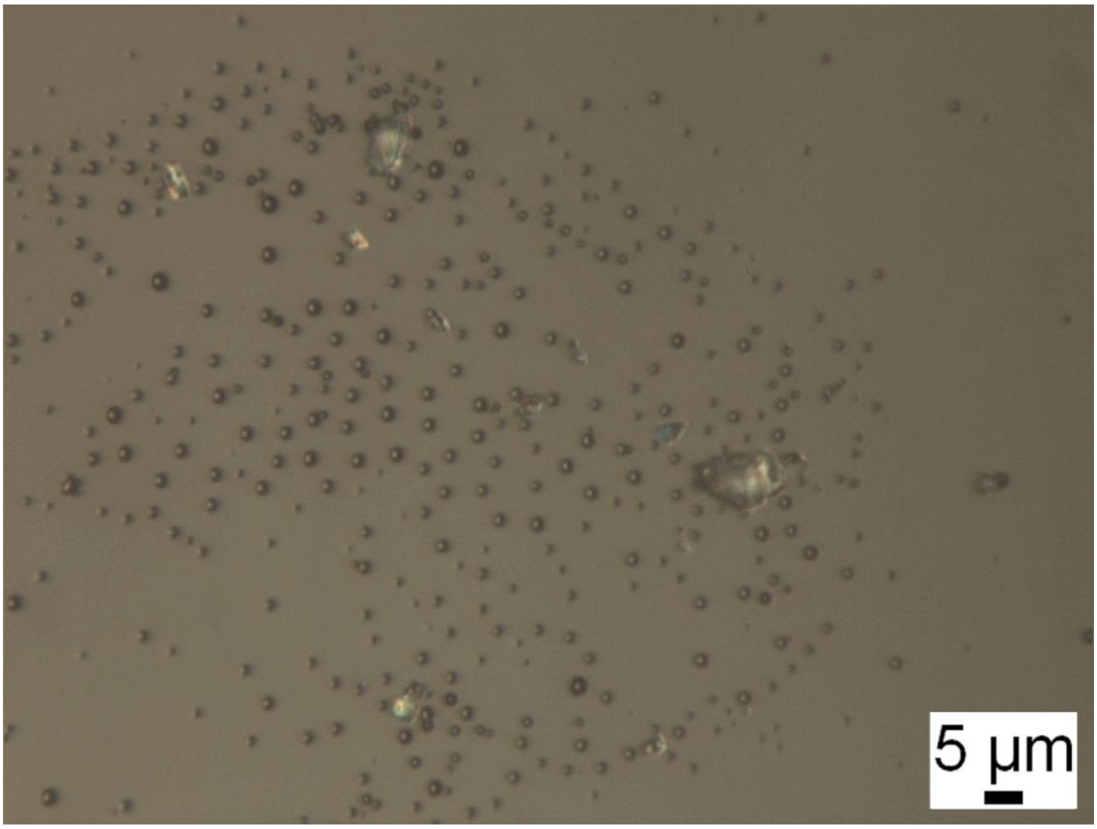

Figure S26 The optical microscopy image of the blending system.

Discussion S4: As shown in **Figure 21-26**, imaging under an optical microscope or SEM showed clearly shaped particles of polymer and inorganic particles, while the differential imaging effect towards polymer and organic particles was relatively poor when compared with that of FM, owing to the poor contrast of these two kinds of particles under the optical microscope or SEM.

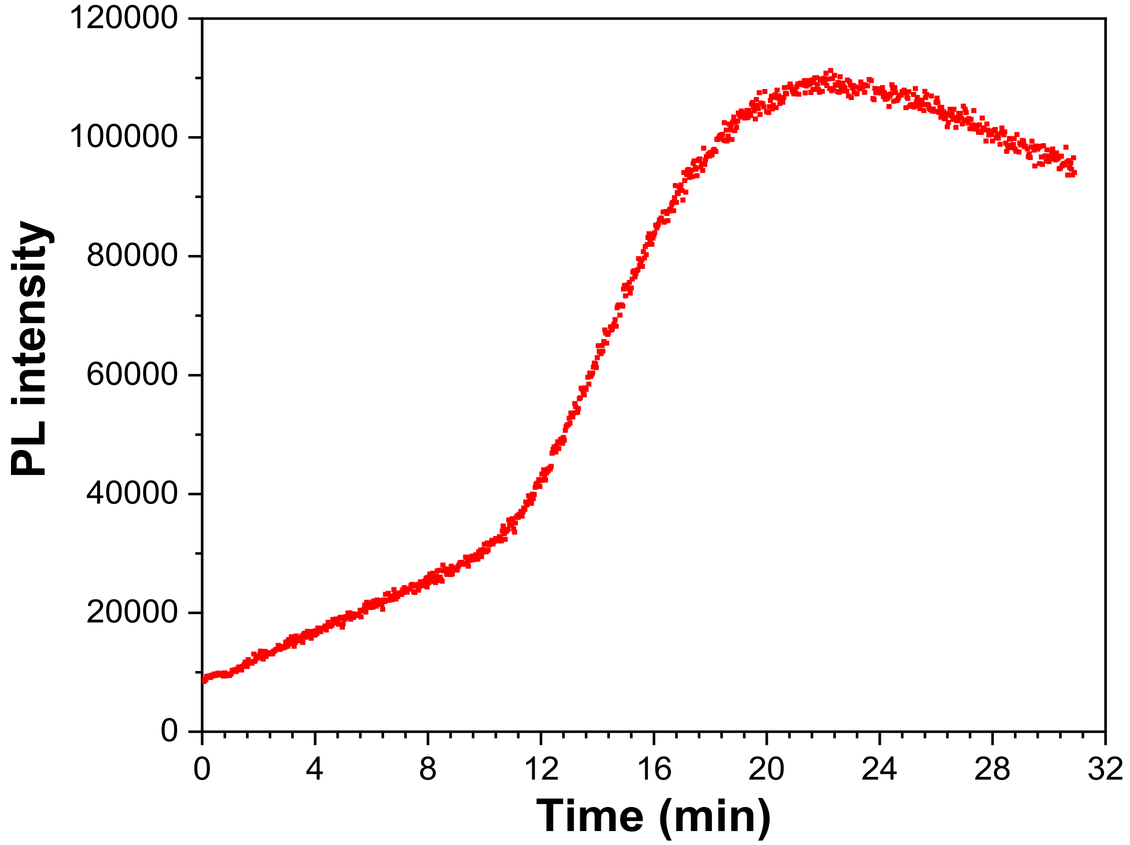

Figure S27 The PL dynamic spectrum of the film formation process under continuous monitoring (photo-bleaching monitored).

Discussion S5: The continuous monitoring of PL signals under the fluorescence spectrometer caused the bleaching of TPE-4S-Na, as evidenced by the decrease in PL intensity after 22 min. Therefore, the excitation UV light was shielded to obtain the discontinuous signals of PL, as shown in Figure 3b; no bleaching was observed thereafter.

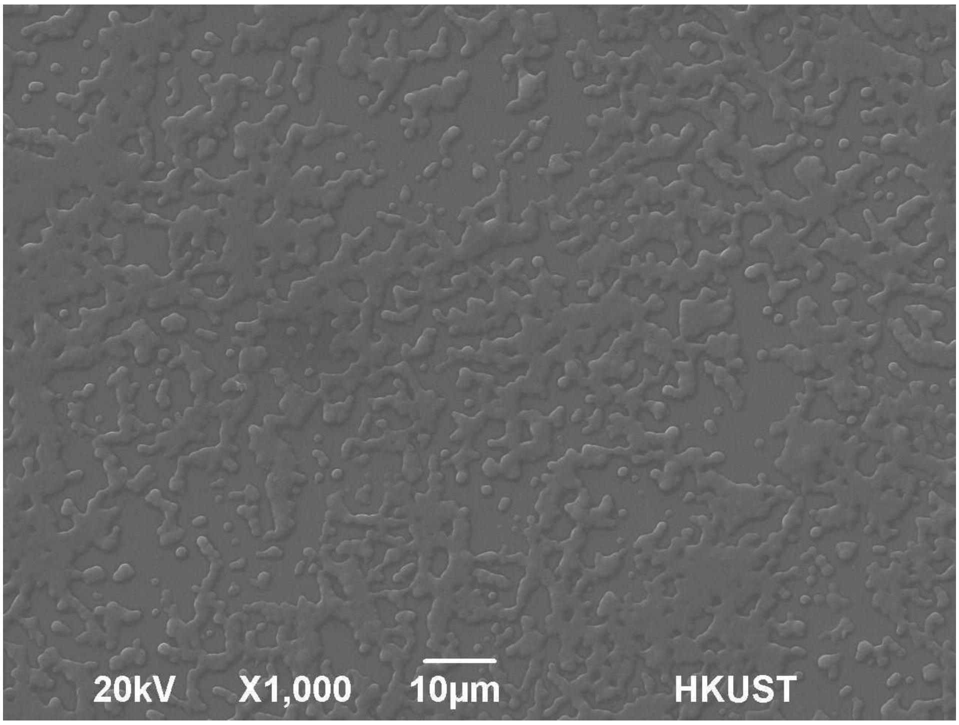

Figure S28 The SEM image of CO2-PUD emulsion particles dried at RT (×1000, the sample was diluted with water 2,000 times).

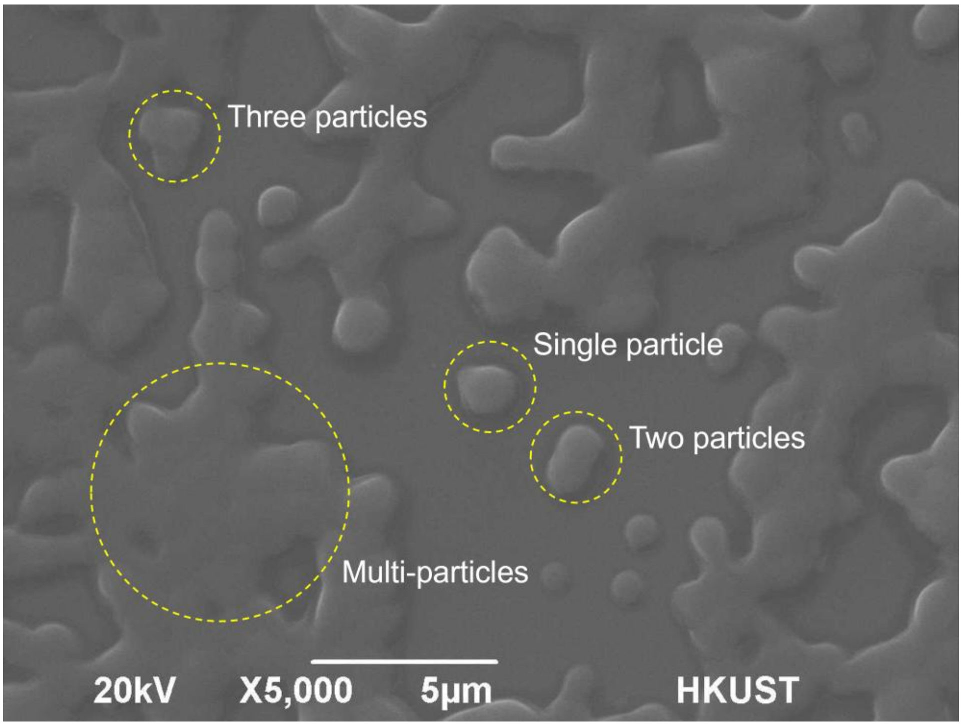

Figure S29 The SEM image of CO_2-PUD emulsion particles dried at RT (×5000, the sample was diluted with water 2,000 times).

Discussion S6: The morphology of individual emulsion particles, as well as configurations of two, three, and multiple emulsion particles, can be clearly observed in the SEM image. However, once more than two emulsion particles aggregate, their contours disappear and cannot be discerned in the SEM image. In contrast, FM images can clearly delineate the boundaries of particle fusion and the capillary regions, thanks to the traces left by AIEgen during the drying process. Therefore, FM is more suited for monitoring the dynamic fusion process of emulsion particles compared to SEM.

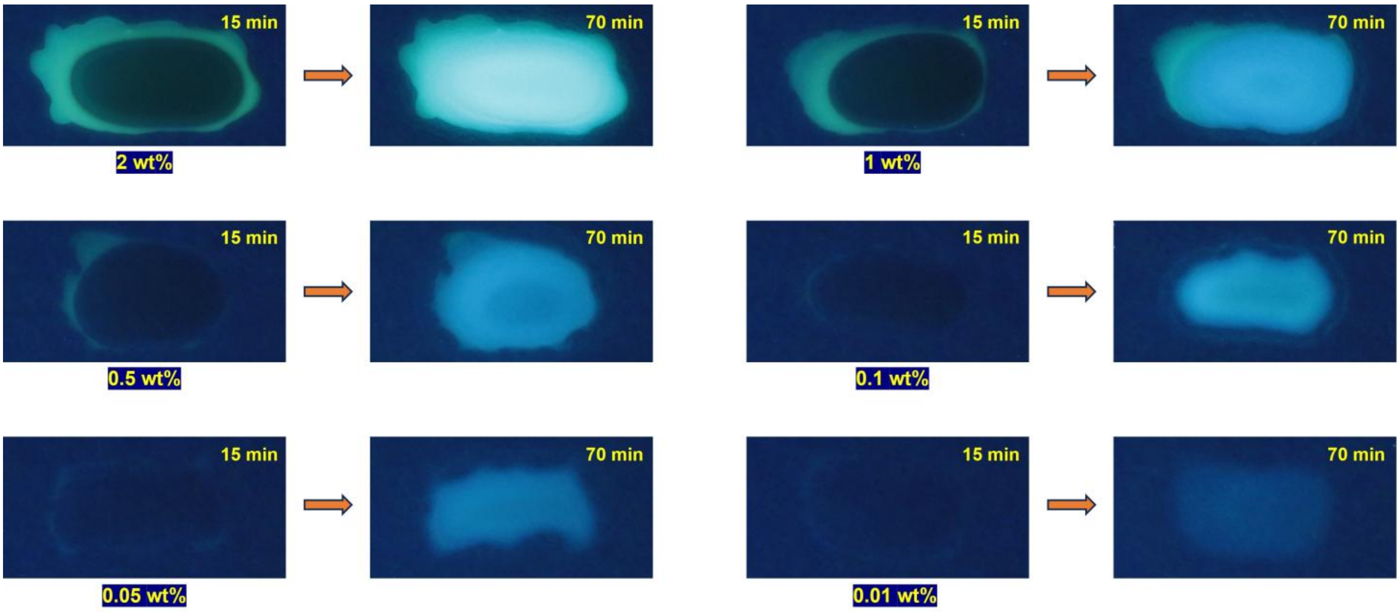

Figure S30 The image of CO_2-WPU with different wt% of TPE-4S-Na under UV light before and after drying.

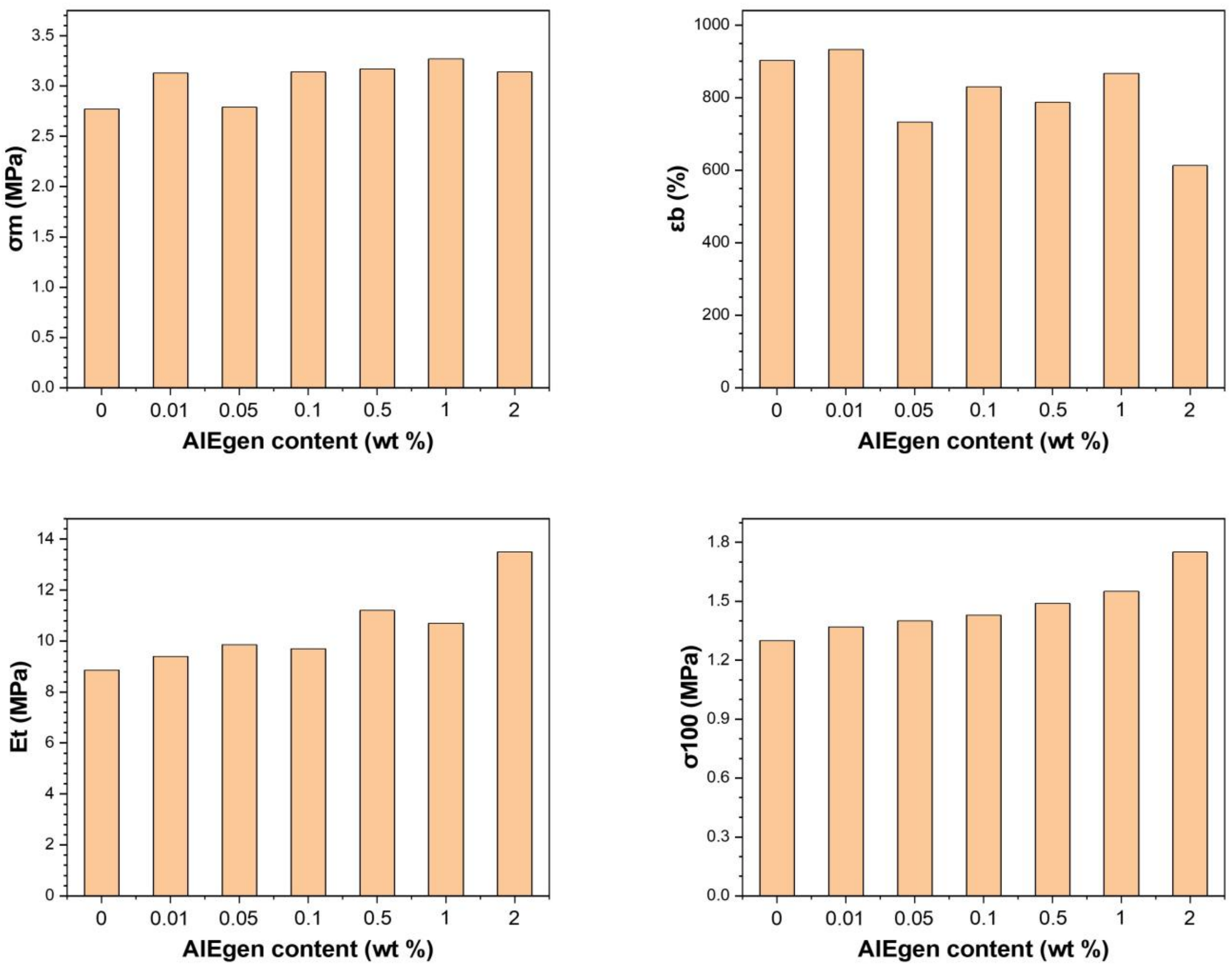

Figure S31 The mechanical performance of dried CO_2-WPU film with the addition of different weights of TPE-4S-Na.

Discussion S7: As shown in Figure **30**, different weights of TPE-4S-Na were added into CO_2-WPU to check the imaging output under UV light. When the addition amount was as low as 0.05 wt%, the high contrast in PL before and after drying indicates that this addition amount was still suitable for monitoring film formation in polymer emulsion.

In addition, the influence of added TPE-4S-Na on the mechanical performance of the dried polymer film was also investigated (Figure S31). Tensile strength (σ_m) and strength at 100% elongation (σ_{100}) increased when more TPE-4S-Na was introduced. As for elastic modulus (E_t), a similar trend was observed. While for flexibility of elongation at break (ε_b), no specific trend was observed. In conclusion, the addition of TPE-4S-Na resulted in an enhanced mechanical strength of the dried polymer film.

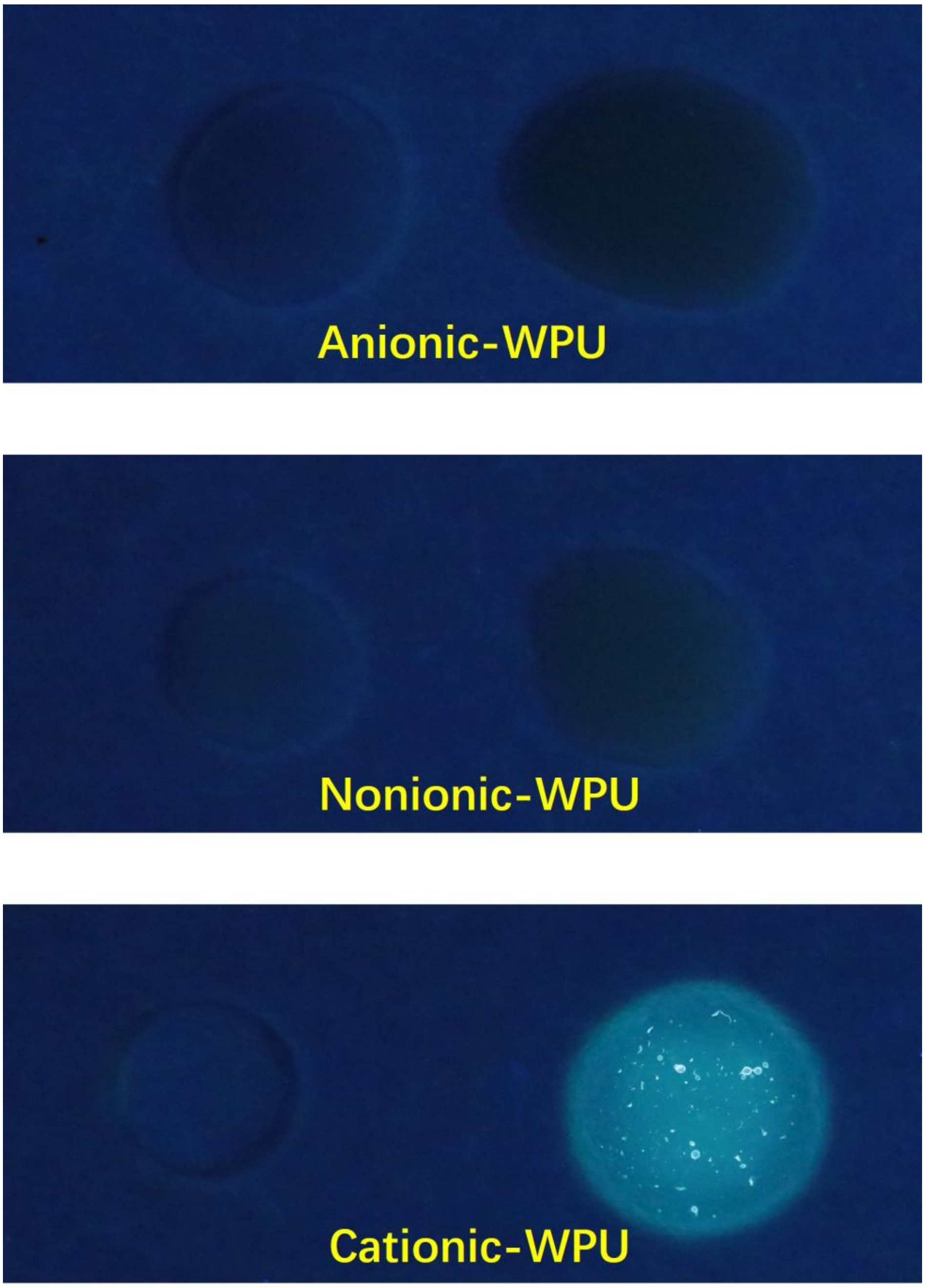

Figure S32 The image of anionic, nonionic, and cationic CO_2-WPU under UV light before (left) and after (right) addition of TPE-4S-Na.

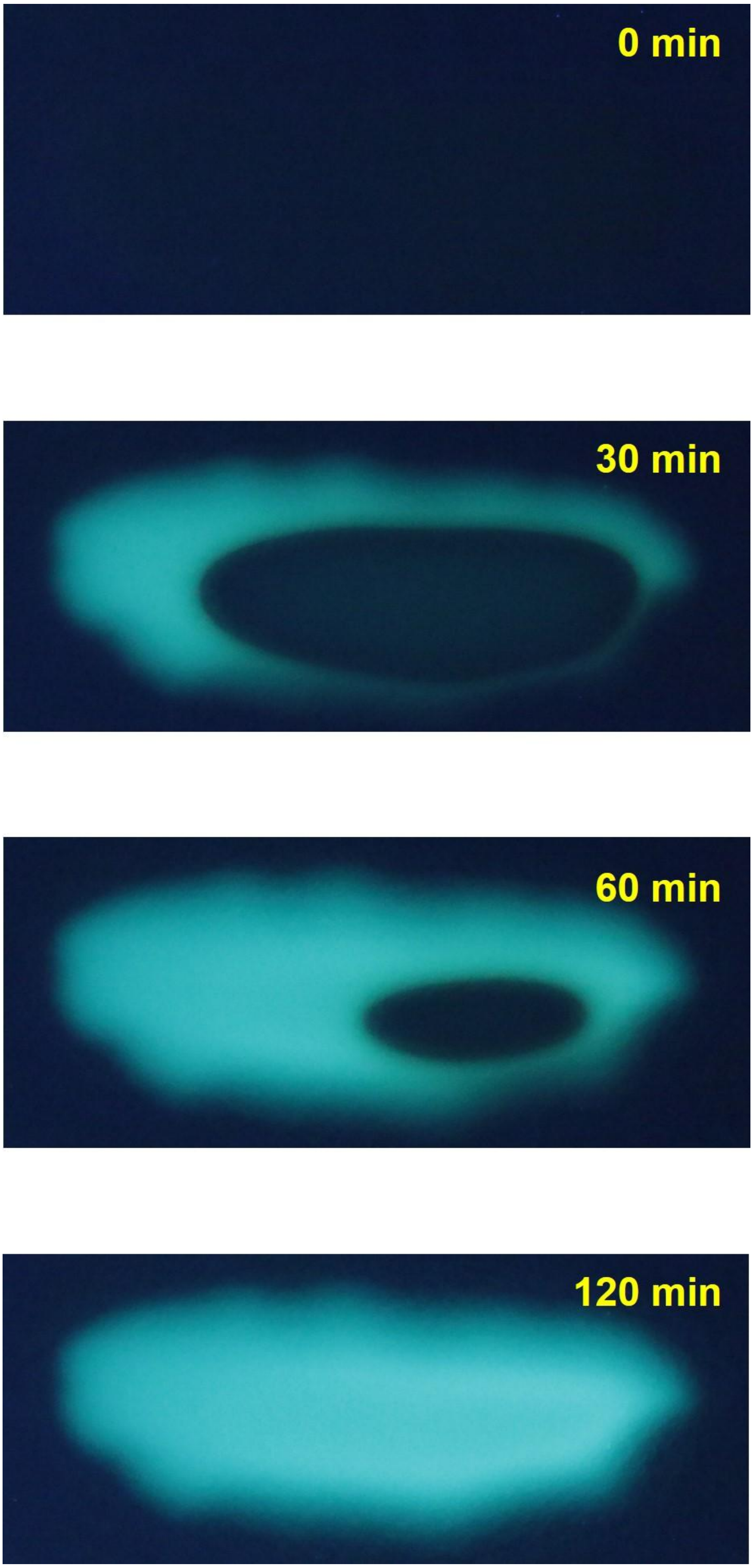

Figure S33 The image of nonionic CO_2-WPU with TPE-4S-Na under UV light before and after drying.

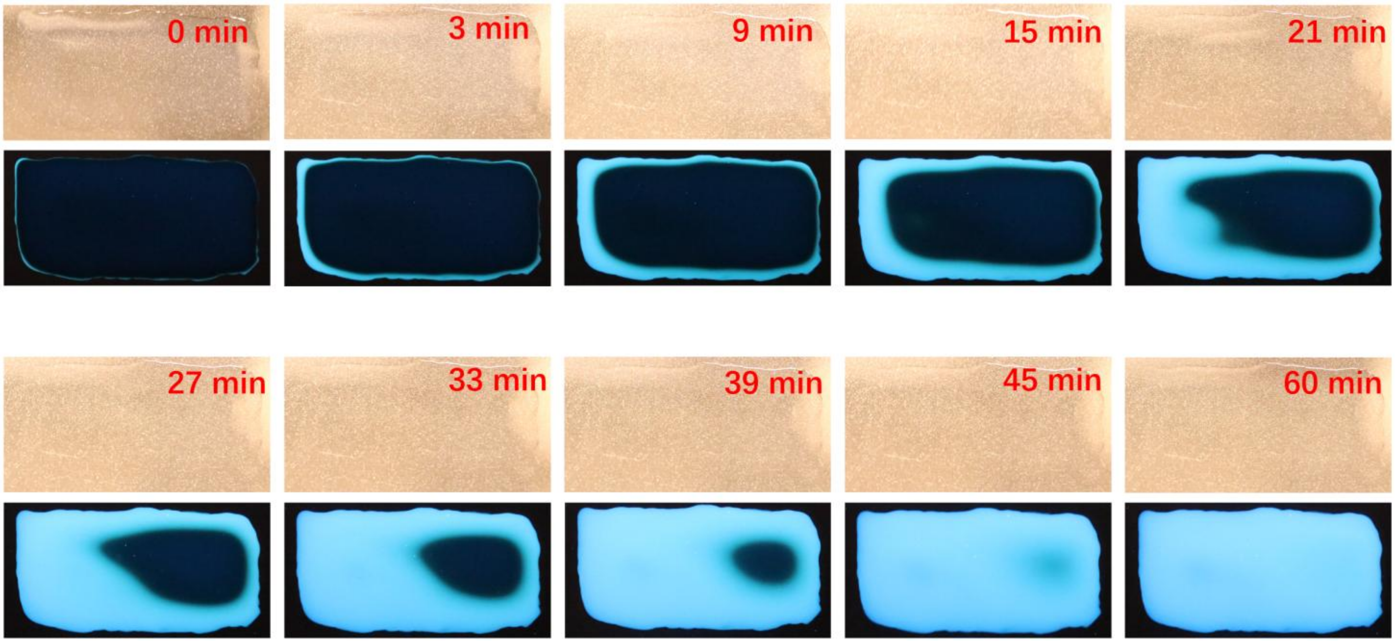

Figure S34 The comparison of the film formation process under daylight (upper) and UV light (lower) for industrialized polyacrylate emulsion.

Discussion S8: According to the type of emulsifier agent, waterborne polyurethane was divided into three types: the anionic, the non-ionic, and the cationic resin. Here, three different types of CO_2-WPU were synthesized, and 1 wt% of TPE-4N-Na was added to test the stability of the emulsion. As shown in **Figure S32**, the anionic and non-ionic CO_2-WPU showed high stability after the addition of TPE-4N-Na aqueous solution, while demulsification was observed for cationic CO_2-WPU. Fortunately, most of the polymer emulsion was anionic type, so the influence of failure in cationic polymer emulsion on the application of AIE-FFM was controllable, and maybe another type of AIE gen may be developed to meet the need for anionic polymer emulsion. The poor stability of cationic CO_2-WPU was attributed to the destruction of the base-acid pair that formed by the combination of ammonium and chloride anion. According to **Figure S33**, AIE-FFM was also feasible in the non-ionic CO_2-WPU.

Despite waterborne polyurethane, polyacrylate emulsion is another kind of the most frequently used polymer emulsion. The AIE-FFM strategy was also applied for the film formation process of industrialized polyacrylate emulsion in **Figure S34**, showing similar feasibility to that of CO_2-WPU. In addition, the monitoring was simultaneously carried out under daylight. The images indicated the much higher contrast of the film formation process under UV light.

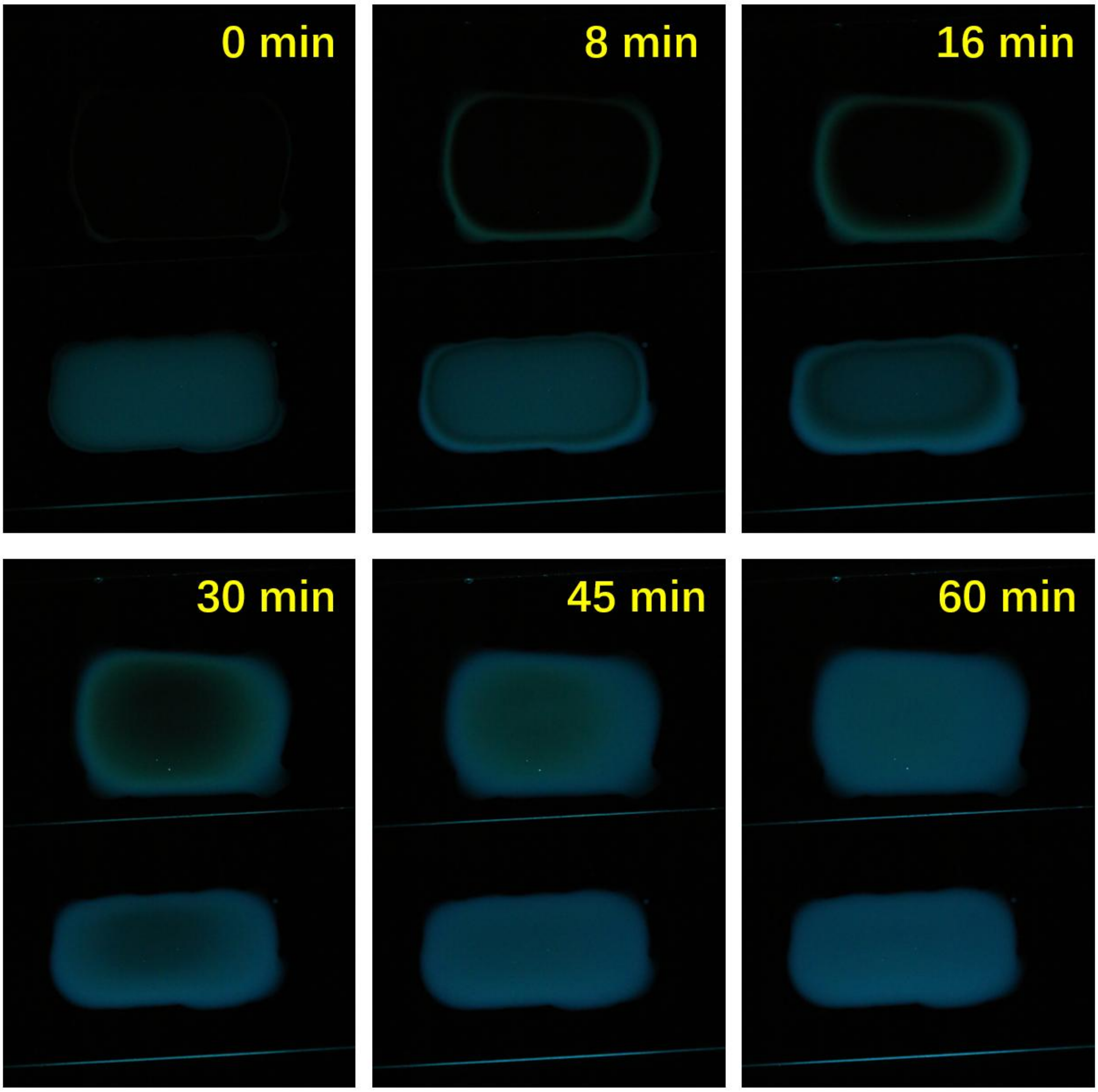

Figure S35 The comparison of TPE-4S-Na (upper) and TPE-4COONa (lower) for monitoring of film formation process for CO_2-WPU.

Discussion S9: The TPE-4COONa showed relatively strong PL in CO_2-WPU emulsion, which may be attributed to the poorer solubility in water when compared with TPE-4S-Na. Thus, TPE-4S-Na showed higher contrast in the monitoring of the film formation process of polymer emulsion.

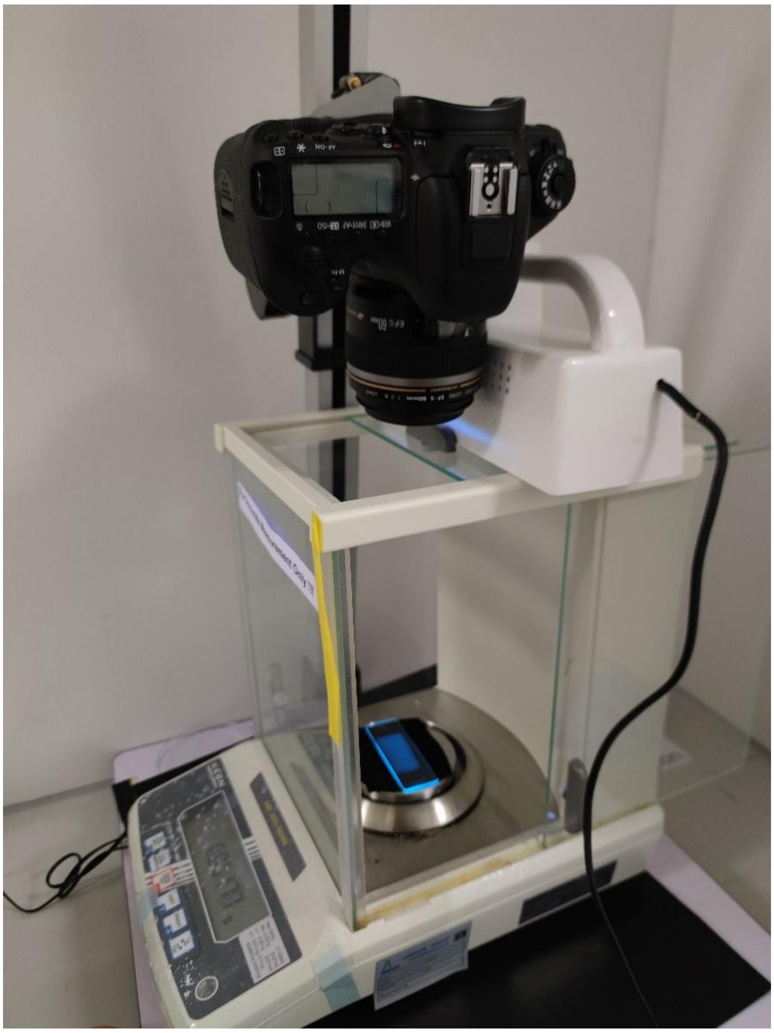

Figure S36 The image of the system that simultaneously monitors both weight and fluorescence changes.

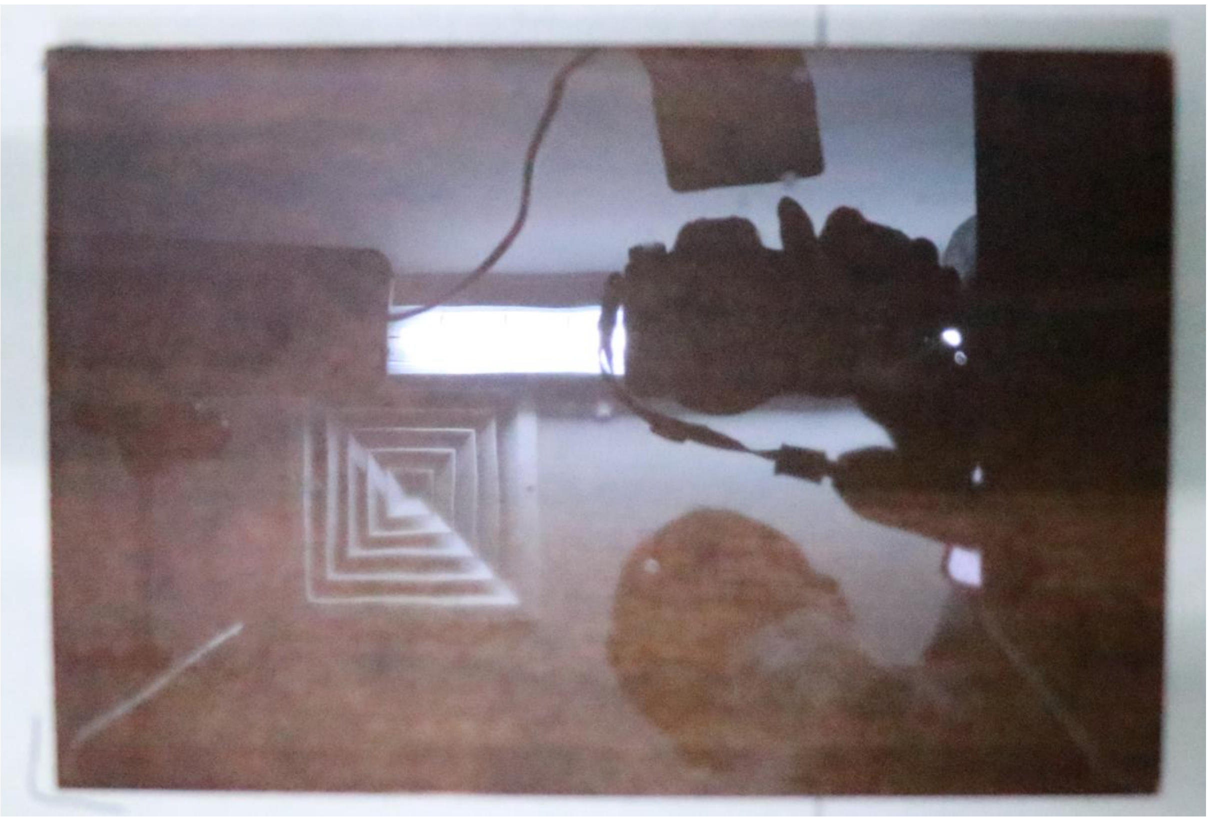

Figure S37 The image of wet film on a wooden board taken under daylight.

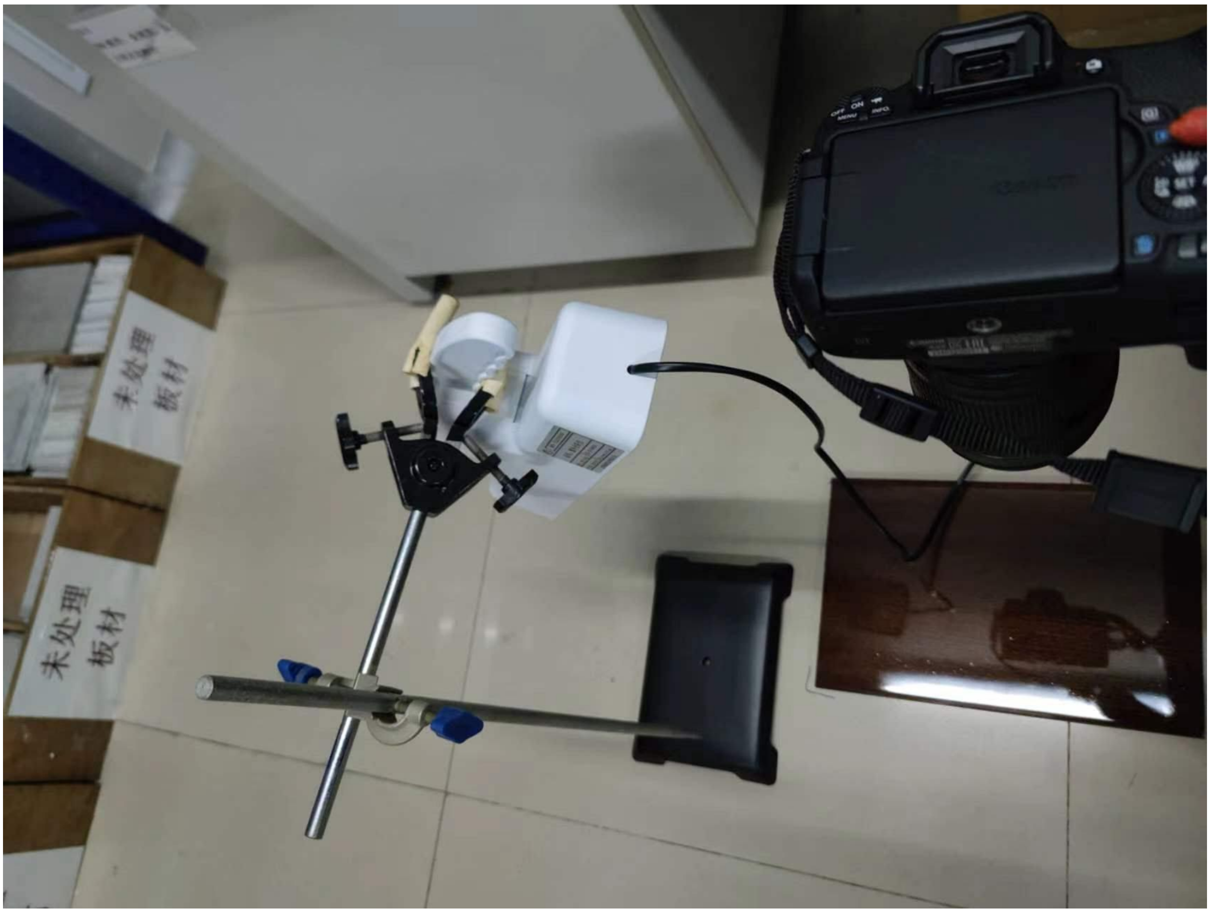

Figure S38 The simple monitoring system in coatings industry.

5. Supplementary references

1. Zhao, Y. K., Gao, Z. Z., Wang, H., Zhang, D. W. & Li, Z. T. Self-assembly of supramolecular polymers in water from tetracationic and tetraanionic monomers in water through cooperative electrostatic attraction and aromatic stacking. *Chinese Chem. Lett.* **30**, 127-130, doi:10.1016/j.cclet.2018.10.016 (2019).

2. Huang, M. F., Cao, L. H. & Zhou, B. A solvent-controlled photoresponsive ionic hydrogen-bonded organic framework for encryption applications. *Chem. Commun.* **60**, 3437-3440, doi:10.1039/d4cc00701h (2024).

3. Wang, J. et al. Waterborne polyurethanes from CO_2 based polyols with comprehensive hydrolysis/oxidation resistance. *Green Chem.* **18**, 524-530, doi:10.1039/c5gc01373a (2016).
